# Dynamic EBF1 occupancy directs sequential epigenetic and transcriptional events in B-cell programming

**DOI:** 10.1101/gad.309583.117

**Published:** 2018-01-15

**Authors:** Rui Li, Pierre Cauchy, Senthilkumar Ramamoorthy, Sören Boller, Lukas Chavez, Rudolf Grosschedl

**Affiliations:** 1 Department of Cellular and Molecular Immunology, Max Planck Institute of Immunobiology and Epigenetics, 79108 Freiburg, Germany;; 2 International Max Planck Research School for Molecular and Cellular Biology, Max Planck Institute of Immunobiology and Epigenetics, 79108 Freiburg, Germany;; 3 Division of Pediatric Neurooncology, German Cancer Research Center, 69120 Heidelberg, Germany;; 4 Department of Medicine, Division of Medial Genetics, School of Medicine, University of California at San Diego, La Jolla, California 92093, USA

**Keywords:** EBF1, Pax5, IRF4, B-cell programming, chromatin, DNA methylation

## Abstract

B-cell fate determination requires the action of transcription factors that operate in a regulatory network to activate B-lineage genes and repress lineage-inappropriate genes. However, the dynamics and hierarchy of events in B-cell programming remain obscure. To uncouple the dynamics of transcription factor expression from functional consequences, we generated induction systems in developmentally arrested *Ebf1*^−/−^ pre-pro-B cells to allow precise experimental control of EBF1 expression in the genomic context of progenitor cells. Consistent with the described role of EBF1 as a pioneer transcription factor, we show in a time-resolved analysis that EBF1 occupancy coincides with EBF1 expression and precedes the formation of chromatin accessibility. We observed dynamic patterns of EBF1 target gene expression and sequential up-regulation of transcription factors that expand the regulatory network at the pro-B-cell stage. A continuous EBF1 function was found to be required for *Cd79a* promoter activity and for the maintenance of an accessible chromatin domain that is permissive for binding of other transcription factors. Notably, transient EBF1 occupancy was detected at lineage-inappropriate genes prior to their silencing in pro-B cells. Thus, persistent and transient functions of EBF1 allow for an ordered sequence of epigenetic and transcriptional events in B-cell programming.

## Abstract (precis)

Li et al. show in a time-resolved analysis that EBF1 occupancy coincides with EBF1 expression and precedes the formation of chromatin accessibility. They observed dynamic patterns of EBF1 target gene expression and sequential up-regulation of transcription factors that expand the regulatory network at the pro-B-cell stage.

The hematopoietic system is a well-studied paradigm for the differentiation of pluripotent stem cells to generate a large variety of effector cells. Differentiation of hematopoietic stem cells via multipotent progenitors generates lineage-committed cells that mature further into specialized effector cells. This process is accompanied by successive lineage restrictions in which cells lose the potential to adopt distinct cell fates (Nutt and Kee 2007; Boller and Grosschedl 2014; Rothenberg 2014). B lymphopoiesis involves lymphoid-biased multipotent progenitors (LMPPs, also referred to as MPP4) that differentiate to common lymphoid progenitors (CLPs). The heterogeneous CLP population includes B-cell-biased lymphoid progenitors (BLPs) and all lymphoid progenitors (ALPs) that retain the potential to generate B and T cells, natural killer cells, and lymphoid dendritic cells (Kondo et al. 1997; Adolfsson et al. 2005; Inlay et al. 2009). Differentiation of multipotent progenitors to lineage-committed cells depends on multiple changes in the transcriptional and epigenetic states of the cells. In particular, multilineage priming of *cis*-regulatory sequences in progenitors has been implicated in setting a permissive chromatin state that facilitates the binding of lineage-specific transcription factors (Hu et al. 1997; Laslo et al. 2006; Heinz et al. 2010; Mercer et al. 2011). Lineage-instructive transcription factors have been proposed to confer de novo chromatin accessibility, and the combinatorial action of transcription factors has been associated with the establishment of lineage-specific programs of gene expression (Singh et al. 2007; Miyazaki et al. 2014; Rothenberg 2014; Iwafuchi-Doi and Zaret 2016). Lineage programming also requires the shutdown of alternative lineage potential in a process termed lineage commitment (Nutt and Kee 2007; Boller and Grosschedl 2014).

Programming of the B-cell lineage requires a complex regulatory network of transcription factors in which synergy and cross-antagonism between multiple transcription factors ensure the robustness of establishing B-cell identity and a shutdown of alternative lineage potential (Boller and Grosschedl 2014; Rothenberg 2014). Specifically, the transcription factors PU.1 and E2A (*Tcf3*) have been shown to be involved in B-lineage priming (Bain et al. 1994; Zhuang et al. 1994; DeKoter and Singh 2000; Heinz et al. 2010; Mercer et al. 2011). E2A also collaborates with FoxO1 to activate the expression of EBF1, which enhances the expression of E2A and FoxO1 and activates the expression of Pax5 (Lin et al. 2010; Mansson et al. 2012). Although these transcription factors are all required for the activation of B-lineage-specific genes, EBF1 appears to have a lineage-instructive function. The forced expression of EBF1 in hematopoietic stem cells or progenitors enhances the generation of B-lineage cells, and the ectopic expression of EBF1 in PU.1- or Ikaros-deficient progenitors allows them to overcome their block of early B lymphopoiesis (Medina et al. 2004; Pongubala et al. 2008; Reynaud et al. 2008; Zandi et al. 2008; Banerjee et al. 2013). Conversely, the targeted *Ebf1* gene inactivation results in a complete block of pre-pro-B-to-pro-B-cell differentiation (Lin and Grosschedl 1995). In addition to its role in establishing the B-cell fate, EBF1 collaborates with Pax5 to regulate the maintenance of B-cell identity. Inactivation of *Ebf1* in committed pro-B cells allows for lineage conversion to T and innate lymphoid cells (Nechanitzky et al. 2013). Moreover, EBF1 was found to repress transcription factor genes that specify alternative lineages (Pongubala et al. 2008; Banerjee et al. 2013; Nechanitzky et al. 2013), whereas Pax5 also represses genes encoding receptors that respond to alternative lineage-promoting signals (Revilla et al. 2012). Therefore, EBF1 and Pax5 appear to govern a double-lock mechanism in enforcing B-lineage identity.

In addition to these functions of EBF1 in regulating gene expression, EBF1 has been implicated in changing the epigenetic landscape (Maier et al. 2004; Treiber et al. 2010; Boller et al. 2016). Expression of EBF1 in multipotent *Ebf1*-deficient progenitors results in the appearance of B-lineage-specific chromatin accessibility and DNA demethylation as determined by genome-wide analysis of DNase I hypersensitivity and CpG methylation (Boller et al. 2016). The C-terminal domain (CTD) of EBF1 was found to allow EBF1 binding at sites in naïve chromatin that lack co-occupancy by other transcription factors, suggesting that this domain confers on EBF1 the ability to act as a pioneer factor. Notably, the function of the CTD of EBF1 was required for the regulation of genes involved in the B-cell versus T-cell fate choice (Boller et al. 2016). However, this analysis did not allow for an assessment of the order and the dynamic progression of epigenetic and transcriptional events.

In the present study, we used induction of EBF1 expression in multipotent progenitors to examine the time course of events underlying EBF1-mediated B-cell programming. We found that the occupancy of EBF1 precedes the formation of chromatin accessibility and changes in gene transcription. Moreover, we observed a sequential expression of B-cell-specific transcription factors that can account for distinct temporal patterns of gene activation and repression during B-cell specification and commitment. Finally, we show that the silencing of many lineage-inappropriate genes at the pro-B-cell stage is preceded by a transient EBF1 occupancy. Thus, our time-resolved analysis of EBF1 function in B-cell programming revealed dynamic alterations of chromatin and sequential changes of regulatory states involved in the activation of B-cell identity genes and repression of lineage-inappropriate genes.

## Results

### Dynamic expression of transcription factors during EBF1-initiated B-cell programming

**Figure 1.**
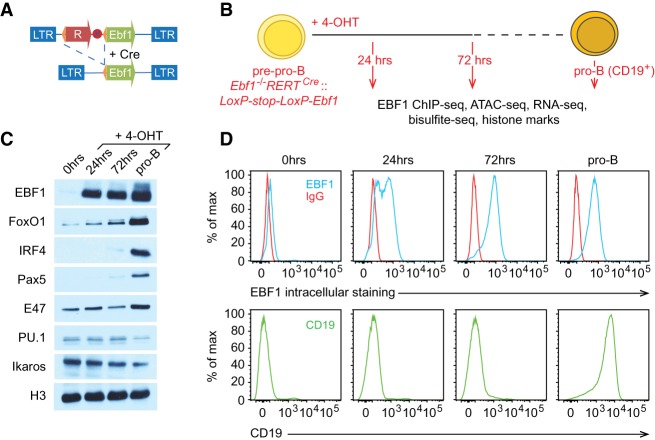
Generation of an inducible EBF1 expression system in *Ebf1*^−/−^ pre-pro-B cells. (*A*) Schematic presentation of a 4-OHT-inducible retroviral EBF1 expression cassette. The retroviral *loxP-Stop-loxP-Ebf1* construct contains a dsRed or tailless mCD8a reporter gene (R) and stop codon (red circle labeled with X) cassette that is flanked by *loxP* sites (orange triangles) and followed by an *Ebf1* cDNA (green box). (LTR) Long terminal repeat. (*B*) Sorted pre-pro-B cells from the fetal livers of *Ebf1*^−/−^ *RERT*^*Cre*^ mice were transduced with *loxP-Stop-loxP-Ebf1* retrovirus. EBF1 expression was induced by the addition of 2 µM 4-OHT, and cells were analyzed at the 24- and 72-h time points and at the CD19^+^ pro-B-cell stage. (*C*) Immunoblot analysis to detect the expression of transcription factors before and after EBF1 induction. (*D*) Flow cytometric analysis of intracellular EBF1 expression and the B-cell surface marker CD19 in *Ebf1*^−/−^ *RERT*^*Cre*^::*LoxP-Stop-LoxP-Ebf1* pre-pro-B cells before and after 4-OHT treatment.

To generate developmentally arrested progenitor cells that can be induced to differentiate into B-lineage cells, we transduced *Ebf1*^−/−^*RERT*^*Cre*^ pre-pro-B cells with a retrovirus carrying a reporter gene and a translational stop codon that are flanked by *loxP* sites and followed by an *Ebf1* cDNA (Fig. 1A). After the addition of 4-hydroxytamoxifen (4-OHT), we collected cells at different time points and performed various genome-wide analyses to assess the dynamics of EBF1 binding, accessible chromatin domains, histone modifications, DNA methylation, and gene expression (Fig. 1B). Immunoblot analysis detected a very low level of EBF1 protein expression at 12 h after 4-OHT addition (data not shown). Within 24 h, EBF1 expression reached a level similar to that observed at the CD19-positive pro-B-cell stage (Fig. 1C). Flow cytometric analysis to detect the surface expression of CD19 on individual cells indicated that virtually no cells acquired this B-cell marker within 3 d after 4-OHT addition (Fig. 1D). Intracellular staining for EBF1 protein by flow cytometry revealed abundant EBF1 expression in the majority of cells at 24 h and in virtually all cells at 72 h (Fig. 1D). Five days after EBF1 induction, 40%–50% of cells acquired CD19 surface expression, and we sorted these cells as pro-B cells for further analysis. Immunoblot analysis of the temporal expression profile of transcription factors implicated in early B-cell differentiation indicated that untreated *Ebf1*^−/−^ pre-pro-B cells carrying the inducible *Ebf1* gene showed weak expression of FoxO1 and E2A (*Tcf3*) but abundant expression of PU.1 (*Spi1*) and Ikaros (*Ikzf1*) (Fig. 1C). However, no expression of Pax5 and IRF4 was detected in the untreated cells. Concomitant with EBF1 expression at 24 h, we observed an up-regulation of FoxO1. At 72 h, we detected very weak expression of Pax5 and IRF4, which was strongly up-regulated in CD19^+^ pro-B cells (Fig. 1C). These results indicate that the induction of EBF1 results in a sequential expression of transcription factors that are known to collaborate with EBF1 in a regulatory network of B-cell fate determination.

### EBF1 occupancy and formation of chromatin accessibility precede DNA demethylation

**Figure 2.**
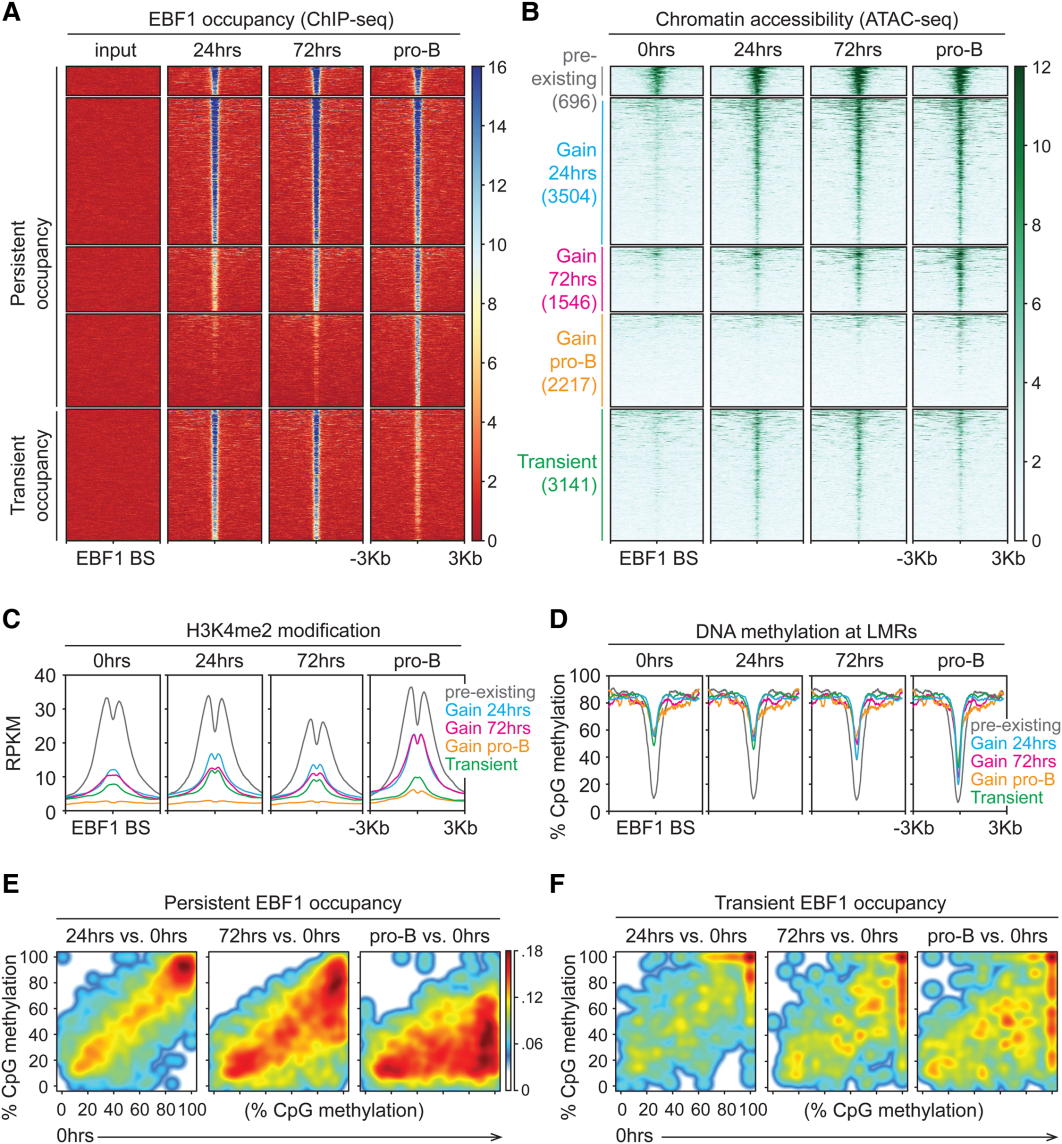
Persistent and transient EBF1 occupancy triggers sequential changes in the epigenome. (*A*,*B*) EBF1 occupancy and chromatin accessibility in *Ebf1*^−/−^*RERT*^*Cre*^::*LoxP-Stop-LoxP-Ebf1* pre-pro-B cells before and after 4-OHT treatment. (*A*) ChIP-seq analysis to detect EBF1 occupancy. A region around ±3 kb of EBF1-binding sites (BS) is shown. The EBF1 peaks are organized into two groups—persistent and transient—in which the peaks detected at 24 and/or 72 h are present or absent at the pro-B-cell stage. The peaks are grouped into five clusters based on the dynamics of EBF1 occupancy and chromatin accessibility. (*B*) ATAC-seq (assay for transposase-accessible chromatin [ATAC] using sequencing) analysis to determine chromatin accessibility. ATAC signals are centered around ±3 kb of EBF1-occupied sites and grouped into five clusters as indicated. The “pre-existing” cluster comprises sites that are accessible before induction and are occupied by EBF1 at 24 h after induction. The other clusters contain regions that are inaccessible before EBF1 induction and gain or lose accessibility coinciding with EBF1 occupancy. The heat map density is represented as RPKM (reads per kilobase per million reads) mean score. (*C*) Dynamics of H3K4me2 modification centered on EBF1-occupied sites of the ATAC clusters described above. (*D*) Dynamics of DNA methylation centered on EBF1-occupied sites that are associated with low methylated regions (LMRs). (*E*,*F*) Cloud maps presenting the levels of CpG methylation in ±100-base-pair windows of persistent (*E*) and transient (*F*) EBF1-occupied sites that are associated with LMRs. The levels at 24 and 72 h after EBF1 induction and at the pro-B-cell stage are compared with the levels before EBF1 induction (0 h).

Previous experiments in which we examined the effects of EBF1 expression on the epigenetic landscape involved the culturing of transduced progenitor cells for >7 d to obtain sufficient numbers of cells for analysis (Boller et al. 2016). The inducible system allowed us to perform ChIP-seq (chromatin immunoprecipitation [ChIP] combined with high-throughput sequencing) analysis to detect EBF1 binding at 24 and 72 h after 4-OHT addition and at the CD19^+^ pro-B-cell stage. During this time course, we detected 7963 EBF1-binding sites that gained occupancy by EBF1 at various time points and remained occupied in pro-B cells. Among these persistently occupied sites, 4200, 1546, and 2217 sites were sequentially gained at 24 h, 72 h, and the pro-B stage, respectively (Fig. 2A). Surprisingly, we found that an additional 3141 sites were efficiently occupied by EBF1 at 24 and 72 h after induction but showed reduced EBF1 occupancy in CD19^+^ pro-B cells (Fig. 2A). Thus, the time-resolved analysis of EBF1 binding not only uncovered dynamic patterns of EBF1 occupancy but also identified a large set of transiently EBF1-occupied sites that had been missed in previous ChIP-seq analyses.

To assess the dynamics of chromatin accessibility during B-cell programming, we performed ATAC-seq (assay for transposase-accessible chromatin [ATAC] using sequencing) analyses at similar time points after EBF1 induction and interrogated the accessible chromatin regions for the presence of EBF1 peaks identified in the ChIP-seq analysis. Consistent with our previous analysis of DNase I hypersensitivity in pre-pro-B and pro-B cells (Boller et al. 2016), we detected pre-existing chromatin accessibility at 696 EBF1-binding sites prior to EBF1 induction (Fig. 2B). The majority of EBF1-binding sites that were occupied at 24 or 72 h also gained chromatin accessibility at these time points. Moreover, the transient occupancy of EBF1-binding sites correlated with a transient gain of chromatin accessibility (Fig. 2B). However, in the cluster that gained EBF1 occupancy at the pro-B-cell stage, we observed low chromatin accessibility. In this cluster, only ∼22% of EBF1 peaks contain canonical EBF1-binding motifs, whereas ∼67%–71% of EBF1 peaks with canonical binding sites were identified in the other clusters (Supplemental Fig. S1A). In addition, while performing a similar clustering approach based on ATAC-seq peaks, we noted that the frequencies of EBF1-occupied sites decreased from ∼90% in the cluster of ATAC-seq peaks at 24 h to ∼42% in the cluster of ATAC-seq peaks that appeared in pro-B cells (Supplemental Fig. S1B). This reduced frequency of EBF1-occupied sites in the accessible chromatin regions of pro-B cells suggests a contribution of other transcription factors to the chromatin landscape at late stages of B-cell programming.

We also analyzed the dynamics of H3K4me2 and H3K27ac modifications and DNA methylation. The cluster of EBF1-binding sites that resides in domains of pre-existing chromatin accessibility showed abundant H3K4me2 and H3K27ac marks prior to and after EBF1 induction (Fig. 2C; Supplemental Fig. S1C). In the cluster of EBF1-binding sites that gains chromatin accessibility at 24 or 72 h after EBF1 induction, we observed a sequential increase of H3K4me2 marks that was even more pronounced at the pro-B-cell stage. We also noted that the overall increase in H3K4me2 intensity was accompanied by a reduction of H3K4me2 as well as H3K27ac marks at the center of EBF1-occupied sites, suggesting that EBF1 binding may involve a local decrease of nucleosome density (Fig. 2C; Supplemental Fig. S1C). In contrast, no obvious active enhancer marks were detected at EBF1-binding sites associated with the “gain pro-B” and “transient” clusters. We also analyzed changes in nucleosome positioning at EBF1-binding sites in pre-pro-B versus pro-B cells. To this end, we analyzed MNase-seq (micrococcal nuclease [MNase] digestion followed by high-throughput sequencing) data of *Tcf3*^−/−^ pre-pro-B cells and *Rag1*^−/−^ pro-B cells (Bossen et al. 2015) at genomic regions that are dynamically occupied by EBF1 (Supplemental Fig. S1D). With the exception of sites residing in the accessible chromatin, impaired MNase digestion was observed at EBF1-binding sites in pre-pro-B cells but not in pro-B cells, suggesting that EBF1 binding coincides with nucleosome remodeling.

To examine the temporal dynamics of DNA demethylation, we performed whole-genome bisulfite sequencing (WGBS) prior to and after EBF1 induction. In particular, we analyzed the CpG methylation status of genomic regions around EBF1-binding sites that are associated with low methylated regions (LMRs). These regions are enriched for enhancers and undergo transcription factor-mediated changes in DNA methylation (Hodges et al. 2011; Feldmann et al. 2013; Ziller et al. 2013). The cluster with pre-existing chromatin accessibility prior to EBF1 induction showed very little DNA methylation. In the cluster that gained chromatin accessibility 24 h after EBF1 induction, we observed a modest loss of DNA methylation at 72 h and a marked loss of methylated CpGs at the pro-B-cell stage (Fig. 2D). In the other clusters, DNA demethylation was also observed predominantly at the pro-B-cell stage. We confirmed this analysis by plotting individual DNA methylation densities for all persistently or transiently occupied EBF1 sites that are associated with LMRs at different stages of EBF1 induction versus preinduction (Fig. 2E,F). The methylation densities for both persistently and transiently occupied EBF1 sites were modestly reduced at 72 h after induction. At persistently occupied EBF1 sites, pronounced demethylation was observed at the pro-B-cell stage (Fig. 2E). In contrast, no further reduction of DNA methylation was observed at transiently occupied sites (Fig. 2F). We also compared the DNA methylation status with a previously published bisulfite sequencing analysis in pre-pro-B and pro-B cells (Benner et al. 2015) and found similar patterns of DNA demethylation (cf. Fig. 2D and Supplemental Fig. S1E). Taken together, these data indicate that EBF1 occupancy and formation of an accessible chromatin domain precedes the loss of DNA methylation.

To examine a potential order in the chromatin binding of other B-cell transcription factors, we performed a digital genomic footprinting analysis during EBF1-induced B-cell programming. To this end, we used the Wellington algorithm that quantifies the protection from Tn5 insertions in the ATAC-seq analysis (Piper et al. 2013). We detected ∼31,000 footprints before EBF1 induction and between ∼41,000 and ∼42,000 footprints at 24 and 72 h after EBF1 induction and at the pro-B-cell stage. We mapped sequence motifs that correspond to binding sites of various B-cell transcription factors to these footprints and determined the temporal dynamics of motif occupancy. Consistent with the ChIP-seq analysis, the digital genomic footprinting revealed almost no occupancy of EBF1 motifs prior to the induction of EBF1 but showed significantly increased occupancy 24 h after EBF1 induction (Supplemental Fig. S2A). Consistent with the time course of Pax5 protein accumulation, the occupancy of Pax motifs was significantly increased at the pro-B-cell stage. A similar increase in the occupancy of Oct motifs was observed in pro-B cells, whereas E-box and FoxO motifs were consistently footprinted throughout the entire time course. As a control, the CTCF motif, known to be largely invariant across cell types (Kim et al. 2007), was also footprinted without significant changes between stages of EBF1 induction.

We also sought to investigate whether EBF1 can recruit other transcription factors to the chromatin after its induction. To determine the co-occurrence of the footprinted motifs with EBF1-occupied motifs or each other in 200-base-pair regions, we performed pairwise co-occurrence enrichment clustering between two time points of EBF1 induction (Obier and Bonifer 2016). This analysis revealed an increasing repertoire of co-occupancy of EBF1 with other transcription factors over the time course of EBF1 induction. Specifically, we observed overall enrichments of co-occurrence of EBF1 footprints with FoxO, E-box, Runx, and Ets footprints at 24 h after EBF1 induction (Supplemental Fig. S2B). Additional co-occurrence of EBF1 and STAT footprints was detected at 72 h, and EBF1 and IRF co-occurring footprints were observed at the CD19^+^ pro-B-cell stage (Supplemental Fig. S2B). In contrast, footprints of Pax and Oct motifs were found predominantly at the pro-B-cell stage, and, notably, these footprints did not co-occur with other motifs examined. As expected, the co-occurrence of CTCF footprints with other footprints was not observed during the time course. Taken together with dynamic expression of transcription factors, these results suggest that the regulatory network in which EBF1 operates involves initially a limited repertoire of transcription factors that is gradually expanded by the sequential expression and chromatin binding of additional transcription factors, including IRF4 and Pax5.

### B-cell programming involves distinct temporal patterns of gene expression

**Figure 3.**
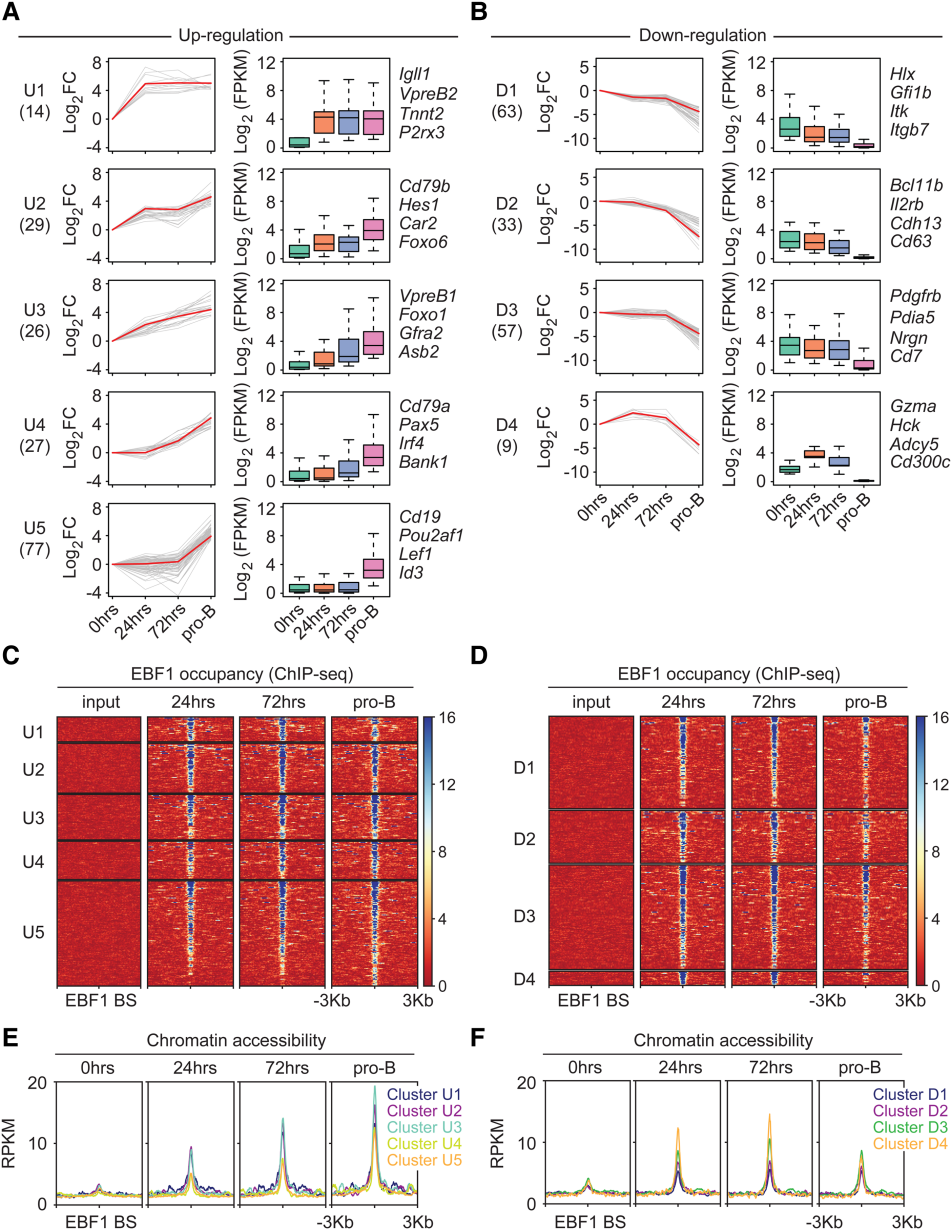
Time-resolved analysis of transcript levels of genes containing EBF1-occupied sites within ±25 kb of transcription start sites before and after EBF1 induction (*A*,*B*). Up-regulated and down-regulated genes that change transcript levels >10-fold between 0 h and the pro-B stage are shown in *A* and *B*, respectively. Genes that are regulated by twofold to 10-fold are shown in Supplemental Figure S4, A and B. Individual transcript levels are shown in Supplemental Table S1 (>10-fold changes) and Supplemental Table S2 (twofold to 10-fold changes). Genes are organized into different clusters based on expression pattern using Short Time-series Expression Miner (STEM) (Ernst and Bar-Joseph 2006). Line plots (*left* panels) and box plots (*right* panels) are used to show fold changes (log_2_ scale) and absolute expression levels (log_2_ scale), respectively. Representative genes of each cluster are listed at the *right*. In each line plot, one representative gene is highlighted in red. (FC) Fold change; (FPKM) fragments per kilobase per million reads. (*C*,*D*) Dynamics of EBF1 occupancy around ±3 kb of EBF1 peaks that are associated with up-regulated genes (*C*) and down-regulated genes (*D*). Clusters correspond to the RNA-seq analysis. (*E*,*F*) ATAC signals around ±3 kb of EBF1 peaks associated with up-regulated genes (*E*) and down-regulated genes (*F*).

To examine the dynamics of EBF1-driven changes in the transcriptome, we performed RNA sequencing (RNA-seq) analysis at different time points after EBF1 induction. Principal component analysis of two biological replicates indicated a marked variance between the samples of uninduced (0 h) and 24-h-induced cells as well as between samples of 24-h-induced and pro-B cells (Supplemental Fig. S3A). In contrast, no significant change in the transcriptome was observed between 24 and 72 h after EBF1 induction. During pre-pro-B-to-pro-B-cell differentiation, the abundance of transcripts from 2425 genes was altered by more than twofold, whereby 1148 of these genes contained persistently or transiently EBF1-occupied sites as determined by ChIP-seq analysis (Supplemental Fig. S3B). We also analyzed a previous data set of dynamic gene expression in progenitor cells in which differentiation was induced by inhibition of Id2 (Mercer et al. 2011). Among the top-ranked 48 activated and 50 repressed genes, 32 activated and 21 repressed genes overlapped with our data set of EBF1-bound and EBF1-regulated genes (data not shown). As weakly deregulated genes tend to dominate the analysis and limit the uncovering of mechanistic principles (Tong et al. 2016), we used a stringent cutoff of >10-fold altered gene expression and a less stringent cutoff of twofold to 10-fold altered expression, yielding groups of 28% and 72% of deregulated genes, respectively (Supplemental Fig. S3C). We subjected both groups of genes to clustering and found multiple clusters with distinct patterns of dynamic gene expression (Fig. 3; Supplemental Fig. S4). A stringent cutoff of >10-fold altered gene expression yielded five and four clusters of up-regulated and down-regulated genes, respectively (Fig. 3A,B; Supplemental Table S1). A less stringent cutoff of twofold to 10-fold altered gene expression yielded a total of 12 clusters (Supplemental Fig. S4A,B; Supplemental Table S2). For further analysis, we focused on the stringent cutoff gene clusters. Cluster U1 includes *Igll1* and *VpreB2*, which were markedly up-regulated at 24 h after EBF1 induction and showed no significant further increase at the pro-B-cell stage (Fig. 3A). Three other clusters (U2–4) are characterized by a gradual increase in transcript levels during the time course analyzed. Cluster U3 includes *Foxo1* and genes that showed sequential up-regulation starting at 24 h after EBF1 induction. Cluster U4 genes such as *Pax5*, *Irf4*, and *Cd79a* initiated their expression at 72 h and were further up-regulated at the pro-B-cell stage. Finally, the largest cluster, U5, includes genes that were activated only at the pro-B-cell stage and may be driven by the expression of Pax5 and/or IRF4.

Likewise, we identified various temporal patterns of down-regulated genes (Fig. 3B; Supplemental Figure S4B). The down-regulation of transcript levels was generally quite modest at 24 and 72 h. However, in virtually all down-regulated gene clusters, the transcript levels decreased sharply at the pro-B-cell stage. In cluster D4, genes were even up-regulated prior to their down-regulation at the pro-B-cell stage. The clusters of down-regulated genes included EBF1-bound and EBF1-regulated genes that are normally expressed in hematopoietic progenitors, such as *Hlx* and *Gfi1b*, or genes that function in alternative cell lineages, including important regulators of T-cell identity (*Bcl11b*) and myeloid cell identity (*Cebpb* and *Irf7*) (Fig. 3B; Supplemental Fig. S4B).

We also interrogated the clusters of gene expression with the data sets of EBF1 occupancy and chromatin accessibility. We observed EBF1 occupancy at most deregulated genes at 24 h (Fig. 3C,D). Notably, the up-regulated genes in clusters U1–5 showed predominantly persistent EBF1 occupancy, whereas a large proportion of down-regulated genes in clusters D1–4 was only transiently occupied and lost EBF1 occupancy at the pro-B-cell stage (Fig. 3C,D). These patterns of persistent and transient EBF1 occupancy were also observed in the clusters of genes that were deregulated by factors of 2–10 relative to uninduced cells (Supplemental Fig. S4C,D). The temporal dynamics of chromatin accessibility in the clusters of up-regulated genes revealed that the patterns of genes expression mostly paralleled those of chromatin accessibility at EBF1-occupied sites (Fig. 3C,E). In particular, clusters U1–3 of up-regulated genes showed a gain of chromatin accessibility at 24 h that was further enhanced at 72 h and at the pro-B-cell stage (Fig. 3C,E). Cluster U4 and U5 genes showed modest chromatin accessibility at 24 and 72 h that was substantially increased at the pro-B-cell stage. The clusters of down-regulated genes showed enhanced accessibility at 24 h that was not significantly changed in pro-B cells, with the exception of cluster D4 (Fig. 3D,F). We also examined the clusters of up-regulated and down-regulated genes as well as genes with unchanged expression for the numbers of associated EBF1-binding sites and their relative distance to transcription start sites (TSSs) (Supplemental Fig. S3D,E). This analysis indicated that EBF1-binding frequency and the distance to the TSS are similar in down-regulated genes and genes with unchanged expression. However, up-regulated genes were enriched for multiple EBF1-binding sites and a close distance between the TSS and the nearest EBF1-occupied site.

Finally, we interrogated the clusters for the dynamics of histone modifications. The clusters of up-regulated genes gained modest levels of H3K4me2 and H3K27ac marks at EBF1-binding sites at 24 h after EBF1 induction, and the levels were markedly enhanced at the pro-B-cell stage (Supplemental Fig. S5A). Conversely, repressive H3K27me3 marks at TSSs were lost at 72 h after induction (Supplemental Fig. S5B). Down-regulated genes showed no significant change in H3K4me2 levels and a modest decrease of H3K27ac levels at EBF1-binding sites throughout differentiation to the pro-B-cell stage (Supplemental Fig. S5C). Consistent with gene down-regulation, H3K27me3 modification at TSSs was increased at the pro-B-cell stage (Supplemental Fig. S5D).

**Figure 4.**
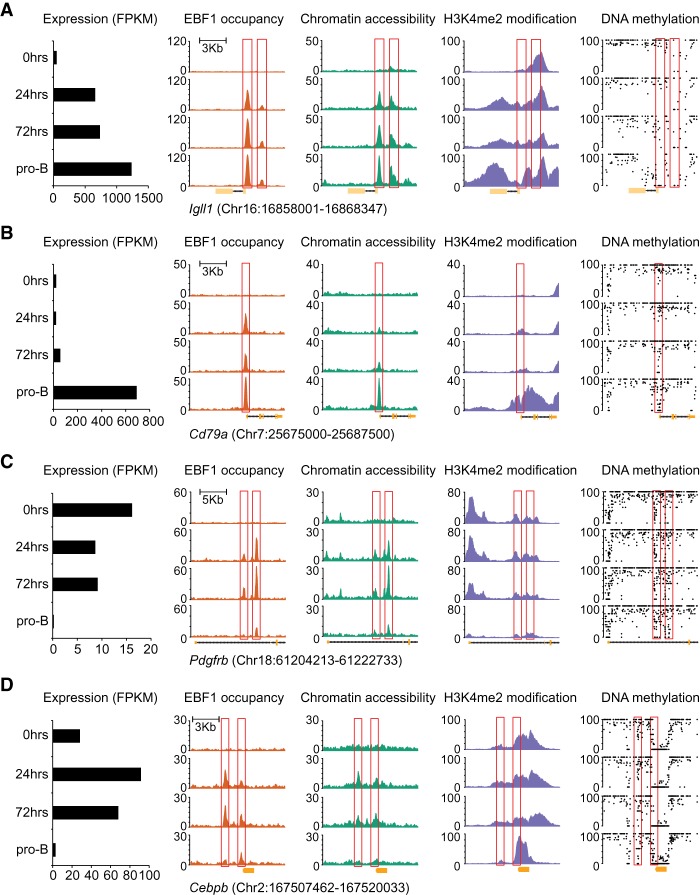
Gene-specific analysis of the dynamics of RNA expression, EBF1 occupancy, chromatin accessibility, H3K4me2 modification, and DNA methylation after EBF1 induction. Representative genes include *Igll1* of cluster U1 (*A*), *Cd79a* of cluster U4 (*B*), *Pdgfrb* of cluster D3 (*C*), and *Cebpb* of cluster D4 (*D*). The positions of EBF1-bound sites are highlighted with red boxes. The scale of the *Y*-axis represents RPKM in ChIP-seq and ATAC-seq tracks and percentage in the DNA methylation tracks, in which each black dot represents one CpG.

We examined the relationship between EBF1 occupancy, chromatin accessibility, H3K4me2 modification, RNA expression, and DNA demethylation also at individual genes representing different clusters of activated and repressed genes (Fig. 4). At the *Igll1* (λ5) locus, we observed a robust gain of EBF1 occupancy and chromatin accessibility at the EBF1-bound site already at 24 h (Fig. 4A). In contrast, DNA demethylation was initiated at 72 h and was completed at the pro-B-cell stage. A similar pattern of EBF1 occupancy was observed for the *Foxo1* gene, although the EBF1-binding site resided already in the accessible chromatin, consistent with the detection of low-level FoxO1 protein and RNA expression in uninduced progenitor cells (Fig. 1C; Supplemental Fig. S6A). The *Cd79a* locus as a representative of cluster U4 was also occupied by EBF1 already at 24 h (Fig. 4B). However, chromatin accessibility was weak at 24 and 72 h and was markedly enhanced at the pro-B-cell stage, concomitant with RNA expression. A similar temporal pattern of EBF1 occupancy, chromatin accessibility, and RNA expression was observed for the *Pax5* locus (Supplemental Fig. S6B). Although *Irf4* showed a temporal pattern of activation similar to that of *Pax5*, we observed EBF1 occupancy at the *Irf4* locus only at 72 h (Supplemental Fig. S6C). As representative down-regulated genes, we analyzed *Pdgfrb* (cluster D3) and *Cebpb* (cluster D4), which were down-regulated in pro-B cells 73-fold and 9.8-fold, respectively (Supplemental Tables S1, S2). In both genes, we detected a transient EBF1 occupancy and chromatin accessibility at 24 and 72 h (Fig. 4C,D). The transient EBF1 occupancy was accompanied by a less than twofold down-regulation of the *Pdgfrb* gene and even up-regulation of the *Cebpb* gene prior to the sharp decrease of RNA expression in pro-B cells.

### EBF1 occupancy precedes the formation of chromatin accessibility

**Figure 5.**
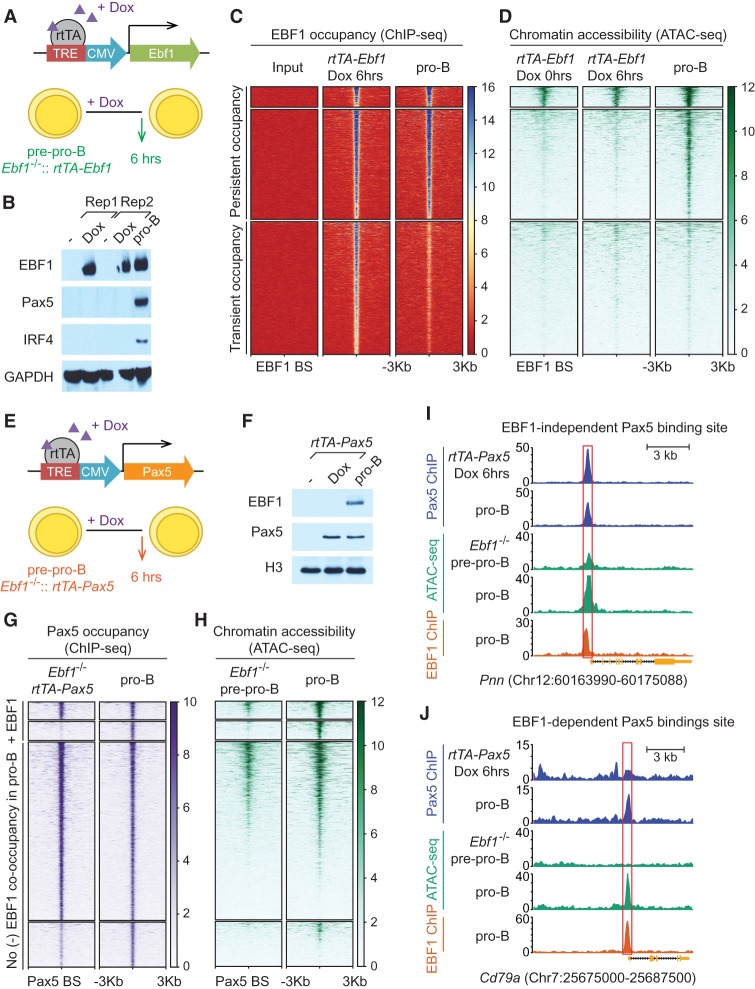
EBF1-induced chromatin accessibility is required for Pax5 occupancy at a set of B-lineage-specific genes. (*A*) Scheme of a “Tet-on”-based doxycycline-inducible EBF1 expression construct, rtTA-Ebf1. *Ebf1*^−/−^ pre-pro-B cells were transduced with *rtTA-Ebf1* retrovirus. Cells were treated with doxycycline (Dox) for 6 h to induce EBF1 expression. (rtTA) Tetracycline-controlled transactivator rtTA-advanced; (TRE) tetracycline response element. (*B*) Immunoblot analysis of cell lysates from *Ebf1*^−/−^::rtTA-Ebf1 pre-pro-B cells at 0 or 6 h after 1 µg/mL doxycycline treatment and from CD19-positive pro-B cells to detect the expression of EBF1, Pax5, and IRF4. (Rep) Replicate. (*C*,*D*) ChIP-seq analysis (*C*) and ATAC-seq analysis (*D*) to detect EBF1 occupancy and chromatin accessibility before (0 h) and after (6 h) doxycycline treatment. EBF1 peaks and ATAC signals are centered around ±3 kb of EBF1-bound sites and grouped into three clusters according to the persistence or transience of EBF1 occupancy and pre-existing or de novo accessibility. (*E*) Scheme of a “Tet-on”-based doxycycline-inducible Pax5 construct (rtTA-Pax5). (*F*) Immunoblot analysis to detect the expression of EBF1 and Pax5 in cell lysates from *Ebf1*^−/−^::rtTA-Pax5 pre-pro-B cells at 0 or 6 h after doxycycline treatment and from pro-B cells. (*G*) Pax5 occupancy in *Ebf1*^−/−^::rtTA-Pax5 pre-pro-B cells (6 h after doxycycline treatment) and in pro-B cells centered on Pax5-bound sites identified in pro-B cells. The *top* two clusters are co-occupied by EBF1 in EBF1-expressing pro-B cells. The *bottom* two clusters lack EBF1 co-occupancy in pro-B cells. (*H*) Chromatin accessibility in *Ebf1*^−/−^ pre-pro-B cells and pro-B cells centered on Pax5-bound sites identified in the pro-B-cell sample. Pax5 peaks and ATAC signals are grouped into four clusters according to the presence or absence of EBF1 co-occupancy in pro-B cells and according to Pax5 occupancy in *Ebf1^−/−^* pre-pro-B cells and EBF1-expressing pro-B cells. (*I*,*J*) Pax5 occupancy in *Ebf1*^−/−^:rtTA-Pax5 pre-pro-B cells (6 h after doxycycline treatment) and in pro-B cells, chromatin accessibility in *Ebf1*^−/−^ pre-pro-B cells and pro-B cells, and EBF1 occupancy in pro-B cells at the EBF1-independent Pax5 target *Pnn* locus (*I*) and EBF1-dependent Pax5 target *Cd79a* locus (*J*).

The 4-OHT-mediated induction of EBF1 expression allowed for abundant EBF1 expression at 24 h but did not yield efficient expression at earlier time points. To determine whether and to what extent EBF1 occupancy precedes chromatin accessibility on a genome-wide scale, we used another induction system in which the *Ebf1* cDNA is linked to a “Tet-on” promoter (Fig. 5A). Addition of doxycycline resulted in abundant EBF1 expression already after 6 h (Fig. 5B). Consistent with the analysis of the 4-OHT-mediated EBF1 induction at 24 h, no expression of Pax5 and IRF4 was observed at 6 h after induction. ChIP-seq analysis to detect EBF1 occupancy indicated that ∼44% of sites that are bound by EBF1 in pro-B cells are already occupied at 6 h (Fig. 5C). In addition, ∼57% of transiently EBF1-occupied sites were already identified at 6 h after EBF1 induction. Interrogation of the ChIP-seq data with an ATAC-seq analysis indicated that the pattern of accessible chromatin domains at EBF1-binding sites was similar before and 6 h after induction, suggesting that EBF1 occupancy precedes the formation of accessible chromatin domains (Fig. 5D).

To examine whether the function of EBF1 as a pioneer transcription factor facilitates the binding of other transcription factors, we examined the ability of Pax5 to bind its targets in the absence of EBF1. Toward this end, we also used the doxycycline-mediated Tet-on induction system in *Ebf1*^−/−^ progenitors to induce expression of Pax5 (Fig. 5E). Six hours after doxycycline addition, Pax5 expression was detected at a level similar to that in pro-B cells (Fig. 5F). ChIP-seq analysis in normal pro-B cells and in doxycycline-induced *rtTA-Pax5 Ebf1*^−/−^ cells identified 5770 Pax5-occupied sites in pro-B cells (Fig. 5G). Of these Pax5-occupied sites, 819 were also co-occupied by EBF1 in pro-B cells. Approximately half of the Pax5-bound sites with neighboring EBF1-binding sites were occupied by Pax5 in the absence of EBF1, and ATAC-seq analysis indicated that many of these sites resided in the accessible chromatin domains (Fig. 5H). However, the other cluster of Pax5-binding sites with co-localized EBF1-binding sites was bound by Pax5 only in EBF1-expressing pro-B cells, suggesting that EBF1 binding is required for Pax5 occupancy. The ATAC-seq analysis indicated that these EBF1-dependent Pax5 sites were inaccessible in progenitor cells and gained accessibility in EBF1-expressing pro-B cells, suggesting that EBF1-dependent changes in chromatin are required for the binding of Pax5. This cluster of Pax5-occupied sites includes many genes playing important roles in B-cell differentiation and pre-BCR signaling, such as *Irf4*, *Cd79a*, *Igll1*, and *Vpreb1* (Supplemental Table S3). As an example for an EBF1-independent Pax5-binding site, the *Pnn* promoter is already accessible in pre-pro-B cells and can be occupied after Pax5 induction in the absence of EBF1 (Fig. 5I). In contrast, the occupancy of Pax5 at the *Cd79a* promoter was observed only in EBF1-expressing pro-B cells in which the site becomes accessible (Fig. 5J). Thus, a functionally important set of genes requires EBF1-induced chromatin accessibility for Pax5 occupancy.

### EBF1 binding is required for *Cd79a* promoter activity and binding of Pax5 and PU.1

**Figure 6.**
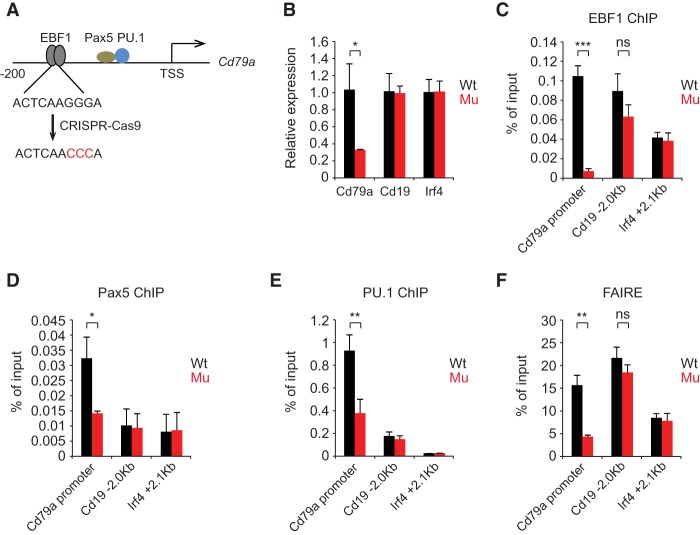
EBF1-induced chromatin accessibility is required for the maintenance of *Cd79a* promoter activity and occupancy by Pax5 and PU.1. (*A*) Scheme of CRISPR/Cas9-mediated mutagenesis of the EBF1-binding sites in the *Cd79a* promoter. The relative positions of transcription factor-binding sites relative to the TSS are indicated. (*B*) qRT–PCR analysis of the indicated genes in wild type and mutant 38B9 cells carrying point mutations in the EBF1-binding site on both alleles. Transcript levels of the *Cd19* and *Irf4* genes served as a control. Transcript levels were normalized. (*C*–*E*) Quantitative ChIP analysis to detect occupancy of EBF1 (*C*), Pax5 (*D*), and PU.1 (*E*) at the *Cd79a* promoter and other regulatory regions of the *Cd19* and *Irf4* genes. (*F*) Chromatin accessibility analysis of EBF1-binding sites at *Cd79a*, *Cd19*, and *Irf4* loci in wild-type and mutant 38B9 cells. (FAIRE) Formaldehyde-assisted isolation of regulatory elements. Error bars represent the standard deviation of three biological replicates. Statistical significance between wild-type and mutant cells was measured by an unpaired two-tail Student's *t*-test. (*) *P* < 0.05; (**) *P* < 0.01; (***) *P* < 0.001.

The *Cd79a* promoter is a well-studied paradigm for an early B-cell-specific promoter that contains a single EBF1-binding site around position −183 and a cluster of nonadjacent binding sites for Pax5, PU.1, and Runx1 between −154 and −47 (Sigvardsson et al. 2002; Maier et al. 2004). To examine whether EBF1 occupancy is required for endogenous *Cd79a* promoter activity and the binding of Pax5 and PU.1, we mutated the EBF1-binding site in both alleles of the endogenous *Cd79a* gene in 38B9 pro-B cells by CRISPR/Cas9-mediated genome editing (Fig. 6A). Mutations in the canonical half-site of the palindromic EBF1-binding site in the *Cd79a* promoter was shown previously to impair binding of EBF1 in vitro (Travis et al. 1993). Quantitative RT–PCR (qRT–PCR) analysis indicated that *Cd79a* RNA expression in the mutated cell line was reduced to 30% of the level found in wild-type pro-B cells (Fig. 6B). As expected, the occupancy of EBF1 was markedly impaired, but we also observed a significant reduction of Pax5 and PU.1 binding (Fig. 6C–E). Moreover, we detected a decrease in the chromatin accessibility around the EBF1-binding site (Fig. 6F). As a control, no changes in the expression, chromatin structure, and EBF1 occupancy of the *Cd19* and *Irf4* genes were observed. Thus, the absence of EBF1 binding in the *Cd79a* promoter results in reduced chromatin accessibility and impaired occupancy by transcription factors.

## Discussion

B-cell programming involves a set of transcription factors that operates in a regulatory network to activate a B-lineage-specific pattern of gene expression and silence genes associated with alternative cell fates (Medina et al. 2004; Lin et al. 2010; Boller and Grosschedl 2014; Rothenberg 2014). To shed light on the functional hierarchy of transcription factors and clarify the temporal order of epigenetic and transcriptional events in B-cell programming, we used multipotent *Ebf1*^−/−^ progenitors in which EBF1 can be induced to mediate pre-pro-B-to-pro-B-cell differentiation. Time-resolved and genome-wide analysis of EBF1 occupancy, chromatin accessibility, DNA methylation, and gene expression indicated that EBF1 occupancy precedes the formation of chromatin accessibility. This analysis also revealed different dynamic patterns of gene expression during EBF1-mediated B-cell programming that correlate with the sequential expression of other transcription factors and changes in the epigenetic landscape. Notably, we also found that EBF1 binds transiently at sites associated with genes that are silenced at the lineage-committed pro-B-cell stage. Thus, B-cell programming involves dynamic transcription factor transactions that allow for a functional hierarchy and “division of labor.”

### Sequence of epigenetic and transcriptional events in EBF1-mediated B-cell programming

The time-resolved analysis of EBF1-mediated B-cell programming allowed us to gain insight into the order of epigenetic and transcriptional events. The fast doxycycline-mediated EBF1 induction system indicated that the majority of EBF1-binding sites is occupied by EBF1 already 6 h after induction. At this time point, we did not detect any significant changes in chromatin accessibility, and only a small fraction of EBF1-occupied sites was found to reside in the accessible chromatin regions. We showed previously that most EBF1-binding sites reside in naïve chromatin and are not associated with active histone marks in progenitor cells (Boller et al. 2016). Moreover, the digital footprinting analysis of transposase-5 cleavage in uninduced *Ebf1*^−/−^ progenitor cells indicated that the EBF1-binding sites are not occupied by a “placeholder” prior to the expression of EBF1. The 6-h time window of EBF1 induction, which results in abundant EBF1 expression after 4 h of doxycycline addition, is also shorter than the cell cycle of progenitor cells, providing strong evidence for a “pioneer function” of EBF1 in binding naïve chromatin regions. However, highly repressive chromatin conformation is nonpermissive for pioneer transcription factor binding (Soufi et al. 2012; Iwafuchi-Doi and Zaret 2014). Consistent with this notion, endogenous EBF1 is unable to bind B-cell targets in nonhematopoietic fibroblastic or mesenchymal OP9 cells (Treiber et al. 2010; S Boller and R Grosschedl, unpubl.). In nonhematopoietic cells, EBF1 binding at B-cell targets may be precluded by a deposition of the repressive histone mark H3K9me3. Megascale domains of H3K9me3 have been shown to restrict the binding of pioneer transcription factors associated with embryonic stem cell pluripotency in somatic cells (Soufi et al. 2012). Moreover, changes in subnuclear localization have been associated with changes in gene expression (Lin et al. 2012; Zullo et al. 2012). Alternatively, incorporation of histone variants in hematopoietic progenitors may facilitate the binding of lineage-specific transcription factors, including EBF1. Thus, the developmental history of cells may dictate which chromosomal regions can be targeted by lineage-specific pioneer transcription factors.

Previously, we had shown that the C-terminal domain of EBF1 is required to allow efficient chromatin binding at sites that contain few other transcription factor-binding sites (Boller et al. 2016). In chromatin regions in which binding sites for other transcription factors, including Pax5 and IRF4, are occupied, the CTD of EBF1 was dispensable for EBF1 occupancy in pro-B cells. In our kinetic analysis, we did not observe any obvious difference in the occupancy of CTD-dependent and CTD-independent sites at 6 h after EBF1 induction (data not shown). At this initial stage of B-cell programming at which neither Pax5 nor IRF4 is expressed, we consider it likely that the CTD is required for the initial occupancy of all EBF1-binding sites that reside in naïve progenitor chromatin.

The time-resolved analysis of B-cell programming allowed us also to gain insight into the order of changes in the chromatin landscape. We found that the formation of accessible chromatin domains around EBF1-binding sites occurred after the detection of EBF1 occupancy. With the exception of a relatively small cohort of EBF1-binding sites that resides in the accessible chromatin in pre-pro-B cells, the majority of EBF1-binding sites acquires accessibility at 24 h or even later. PU.1 has been shown previously to act as a pioneer transcription factor in progenitor cells (Heinz et al. 2010; Barozzi et al. 2014); therefore, we examined PU.1 occupancy in regions of EBF1- and Pax5-binding sites. Using previously published PU.1 ChIP-seq data in *Ebf1*^−/−^ pre-pro-B cells (Heinz et al. 2010), we found that PU.1 occupancy is detected in the cluster of pre-existing chromatin accessibility and in regions in which Pax5 can bind without collaboration with EBF1 (Supplemental Fig. S7A,B).

Changes in the epigenetic landscape after transcription factor binding was also observed for the doxycycline-mediated induction of the basic helix–loop–helix protein NeuroD1 in murine embryonic stem cells (Pataskar et al. 2016). Induced NeuroD1 binding to specific target enhancers results in a subsequent loss of repressive H3K27me3 marks and a gain of H3K27ac marks and target gene expression. The delay in the generation of chromatin accessibility may depend on the pre-existing chromatin state and a slow temporal response of chromatin-modifying enzymes and/or combinatorial action of transcription factors. The slow process of changing chromatin states may help progenitor cells to proliferate before committing to a cell lineage and transit from a mixed-lineage state to the specification of a specific cell fate (Olsson et al. 2016).

The question arises of which mechanisms underlie the generation of accessibility after binding of EBF1. We found that two sets of EBF1-binding sites that are occupied by 24 h of EBF1 induction also gain chromatin accessibility (see Fig. 2B). At this time point, we also detected changes in the transcription of genes that are associated with EBF1-occupied and accessible chromatin regions. The gain of chromatin accessibility could reflect the EBF1-mediated recruitment of chromatin remodeling complexes and/or enzymes that deposit active histone marks. BRG1, a component of the SWI/SNF chromatin remodeling complex, has been implicated in changing the epigenetic landscape in early B-cell differentiation (Gao et al. 2009; Choi et al. 2012; Bossen et al. 2015). In particular, BRG1-associated sites are depleted of nucleosomes during the pre-pro-B-to-pro-B-cell transition (Bossen et al. 2015). BRG1 has been found to associate with the closely related EBF2 protein in adipocytes (Shapira et al. 2017). However, the knockdown of BRG1 does not affect the CTD function of EBF1 (Boller et al. 2016), suggesting that other domains of EBF proteins may be involved in recruiting the SWI/SNF chromatin remodeling complex. Moreover, the different kinetics of chromatin accessibility in various clusters of EBF1-occupied regions and the close association of chromatin accessibility and transcription do not preclude the possibility that a functional cooperation of EBF1 with other transcription factors that are present at early stages of B-cell programming dictates the temporal pattern of transcription and chromatin accessibility.

Our analysis also indicated that DNA demethylation is a later event in B-cell programming and is detected at the pro-B-cell stage. A role of EBF1 in DNA demethylation has been proposed (Maier et al. 2004; Boller et al. 2016). However, the delayed loss of methylated CpGs makes it unlikely that EBF1mediates the recruitment of enzymes of the Tet family. Moreover, the recent analysis of Tet2/Tet3 double deficiency in the B-cell lineage indicates that Tet function is required for immunoglobulin light chain gene rearrangements at the pre-B-cell stage (Lio et al. 2016; Orlanski et al. 2016). Therefore, we consider it likely that general transcription factor occupancy at EBF1-bound regions results in a turnover of DNA methylation (Feldmann et al. 2013).

### Dynamics of B-lineage-associated transcription factors and functional division of labor

In our time-resolved analysis of EBF1-mediated B-cell programming, we found that important B-lineage transcription factors, including FoxO1, Pax5, and IRF4, are sequentially activated by EBF1. These dynamics of transcription factor expression, which may dictate the dynamics of target gene expression, suggest that the regulatory network in which the B-cell transcription factors operate is gradually assembled. EBF1 is functionally connected with E2A and FoxO1 via reciprocal feedback loops that allow for stable initiation of B-cell programming (Mansson et al. 2012; Boller and Grosschedl 2014). The delay in the expression of Pax5 and the silencing of genes associated with alternative cell fates also suggest that the processes of cell fate specification and commitment to the B-cell lineage are linked but temporally separable. Likewise, T-cell specification and commitment involve a regulatory network consisting of Notch, TCF1, GATA3, and Bcl11b that is gradually assembled (Ikawa et al. 2010; Li et al. 2010; Rothenberg 2014; Kueh et al. 2016). A kinetic analysis of the expression of the transcriptional regulator of T-cell commitment, Bcl11b, indicated that the transcription factors TCF1 and GATA3 are expressed several days before they activate the *Bcl11b* gene (Kueh et al. 2016). This time window appears to be needed to “prepare” the *Bcl11b* gene for subsequent expression.

Although many transcription factors work in combination to establish the B-cell lineage program, they appear to accommodate a functional “division of labor.” The analysis of mice carrying targeted deletions of *Tcf3* (E2A), *Ebf1*, *Pax5*, and *Foxo1* indicated that all of these transcription factors are required for early B-cell differentiation (Bain et al. 1994; Urbanek et al. 1994; Zhuang et al. 1994; Lin and Grosschedl 1995; Dengler et al. 2008). However, experiments in which several of these transcription factors were examined for their potential to bypass a differentiation block of Ikaros- or *Ebf1*-deficient progenitors indicated that EBF1, but not E2A or Pax5, can initiate B-cell differentiation (Medina et al. 2004; Reynaud et al. 2008). Moreover, EBF1 can establish lineage-specific chromatin accessibility in the absence of detectable Pax5 expression. A functional hierarchy can be further inferred from the site-specific deletion of the EBF1-binding site in the *Cd79a* promoter, which abrogated EBF1 occupancy but also impaired binding of Pax5 and PU.1 at nonadjacent sites. This observation is similar to the recent analysis of the *Klf4* enhancer in embryonic stem cells in which deletion of the Oct4/Sox2 site reduced chromatin accessibility and prevented the binding of STAT3 and ESRRB (Xie et al. 2017). Conversely, deletion of STAT3- or ESRRB-binding sites impaired *Klf4* expression but did not affect Oct4/Sox2 binding. Thus, a specific set of transcription factors binds at regulatory regions and recruits other transcription factors that link signal transduction and chromatin modification to gene transcription. Likewise, the lineage-specific transcription factors C/EBPα and GATA1 were shown to shift the recruitment of the signal-responsive transcription factor SMAD1 to myeloid- and erythroid-specific regions, respectively (Trompouki et al. 2011). Taken together, these data suggest that the modular assembly of transcription factors allows for a division of labor in controlling complex developmental programs of gene expression.

### The role of transient EBF1 occupancy in gene silencing

An unexpected finding of our genome-wide kinetic analysis of EBF1 occupancy and gene transcription during pre-pro-B-to-pro-B-cell differentiation was the transience of EBF1 occupancy that was very often found to precede gene silencing. Previously, we identified several genes—including *Tcf7*, *Id2*, and *Flt3*—that are down-regulated and occupied by EBF1 at the pro-B-cell stage (Pongubala et al. 2008; Treiber et al. 2010; Boller et al. 2016). In the current kinetic analysis, we identified a much larger number of genes in which silencing at the pro-B-cell stage is preceded by transient EBF1 occupancy and transient chromatin accessibility. Transient EBF1 occupancy was observed in not only the clusters of delayed silencing but also clusters that show an early kinetics of transcriptional down-regulation. Although the repression of several genes at the pro-B-cell stage coincided with the expression and binding of Pax5 (Revilla et al. 2012), the preceding EBF1 occupancy was observed at a larger number of genes. Therefore, these observations raise two questions: First, what is the functional role of transient EBF1 occupancy in gene silencing? Second, how is the transience of EBF1 occupancy achieved?

The transience of EBF1 binding and chromatin accessibility may facilitate the binding of other transcription factors associated with gene repression and/or the recruitment of corepressors, including enzymes that deposit repressive histone marks. Pax5 has been identified as an important repressor of genes associated with alternative cell fates (Nutt et al. 1999; Cobaleda et al. 2007; Revilla et al. 2012). Indeed, many of the transiently EBF1-occupied genes, such as *Cd28*, *Csf1*, *Thy1*, *Id2*, and *Notch1*, are also bound and repressed by Pax5 (Revilla et al. 2012). However, many other silenced genes that were found to be transiently occupied by EBF1 do not contain Pax5-bound sites and may require other transcription factors and/or an EBF1-dependent recruitment of corepressors.

How is the transience of EBF1 binding regulated? One simple mechanism of a developmental transience of transcription factor occupancy involves a replacement by a related but functionally distinct protein that displays a similar or higher binding specificity and/or abundance. For example, the occupancy by FoxO1 in naïve CD4-positive T cells precedes the binding of the structurally related FoxP3 protein during regulatory T-cell specification (Samstein et al. 2012). In this case, FoxO1 acts as a “placeholder” for Foxp3 and helps to generate a chromatin landscape that facilitates subsequent binding of Foxp3.

Another mechanism is the competitive displacement of a transcription factor by an unrelated protein that binds to a distinct but overlapping nucleotide sequence. This mechanism has been shown to account for the displacement of EBF1 from the *Igll1* locus (encoding the surrogate light chain λ5) by the Ikaros-related protein Aiolos at the transition of the pre-BI to the pre-BII stage (Thompson et al. 2007). At this developmental stage, pre-BCR-mediated up-regulation of Aiolos results in the silencing of genes encoding the surrogate light chain components. As the abundance of Aiolos is increased at the late pre-B-cell stage and the abundance of Ikaros is decreased in pro-B cells relative to pre-pro-B cells, we consider it unlikely that the transience of EBF1 occupancy is due to a competitive displacement by proteins of the Ikaros family. Finally, changes in the subnuclear localization of gene loci and association with heterochromatic regions have been implicated in gene silencing and loss of transcription factor occupancy (Reddy et al. 2008; Lin et al. 2012; Zullo et al. 2012).

In conclusion, our time-resolved analysis of EBF1-mediated B-cell programming revealed dynamic functions of EBF1 that may help to establish and coordinate a complex interplay of multiple transcription factors necessary for implementing a complex developmental program of cell fate determination.

## Materials and methods

### Cell culture and retroviral transduction

38B9 pro-B cells were cultured in RPMI-1640 medium supplemented with 10% FCS, 1% PSG, and 50 µM β-mercaptoethanol. To isolate c-Kit^+^ progenitor cells, *Ebf1*^−/−^*RERT*^Cre^ fetal liver cells were stained with the biotinylated c-Kit antibody (BD Biosciences 553353) and purified by streptavidin bead-mediated magnetic-activated cell sorting (MACS) (Miltenyi Biotec). OP9 feeder cells were used to maintain the progenitors in OptiMEM medium supplemented with 4% FCS, 1% PSG, 50 µM β-mercaptoethanol, 10 ng/mL SCF, 10 ng/mL Flt3L, and 5 ng/mL IL-7.

### Cloning and retroviral transduction

Retro-X Tet-on advanced inducible expression system was purchased from Clontech. Ebf1 or Pax5 was cloned to pRetroX-Tight-Pur at NotI and EcoRI sites. rtTA-advanced was cloned to pMys-IRES-GFP at BamHI and EcoRI sites. To generate the Cre-based inducible system, dsRed and eGFP were replaced in pMSCV-loxp-dsRed-loxp-eGFP-Puro-WPRE (Addgene plasmid 32702, kindly provided by Hans Clevers) by tailless *Cd8a* and *Ebf1*, respectively. Retroviral transductions of these plasmids were performed as described (Treiber et al. 2010).

### Flow cytometry

For intracellular staining, cells were fixed and permeabilized with eBioscience transcription factor staining buffer set (Thermo Fisher, 00-5523-00). Cells were stained with anti-EBF1 (peptide purified; BioGenes) or normal rabbit IgG (Cell Signaling, 2729). Alexa fluor 488-conjugated anti-rabbit IgG (Thermo Fisher, A-11034) was used to label the primary antibodies. Cells were analyzed using BD LSRII. The data were processed and visualized with FlowJo. CD19 staining was performed as described (Boller et al. 2016) with APC-coupled anti-CD19 antibody (BD Pharmingen, 550992).

### Immunoblotting and ChIP

Immunoblotting was performed with the following antibodies: anti-EBF1 (BioGenes), anti-Pax5 (Santa Cruz Biotechnology, sc-1974X), anti-IRF4 (Santa Cruz Biotechnology, sc-377383), anti-GAPDH (Calbiochem, clone 6C5), anti-FoxO1 (Santa Cruz Biotechnology, sc11350), anti-E47 (BD Pharmingen, 554077), anti-PU.1 (Santa Cruz Biotechnology, sc-352X), anti-Ikaros (Santa Cruz Biotechnology, sc-13039), and anti-H3 (Abcam, ab1791).

ChIP was performed as described (Boller et al. 2016) with the following antibodies: 4 µg of anti-EBF1 per sample (BioGenes), 4 µg of anti-Pax5 per sample (Santa Cruz Biotechnology, sc-1974X), 4 µg of anti-PU.1 per sample (Santa Cruz Biotechnology, sc-352X), 2 µg of anti-H3K4me2 per sample (Millipore, 07-030), 4 µg of anti-H3K27me3 per sample (Millipore, 07-449), and 2 µg of anti-H3K27ac per sample (Abcam, ab4729). Library preparation and deep sequencing of ChIP samples were performed by the Deep Sequencing Facility at the Max Planck Institute of Immunobiology and Epigenetics. The paired-end reads were mapped to the mouse reference genome (mm9) using Bowtie2 (version 2.3.2) with the default setting (Langmead and Salzberg 2012). The duplicated reads and the reads with low mapping quality were removed using SamTools (Li et al. 2009). The properly mapped reads were used for peak calling by MACS2 (Zhang et al. 2008). The peaks commonly identified in two replicates (if applicable) were extracted for subsequent analysis. DeepTools2 (Ramirez et al. 2016) was used to normalize and visualize the genome-wide data. Transcription factor occupancy and histone modifications at specific loci were visualized using the R package Gviz (Hahne and Ivanek 2016).

### Chromatin accessibility analysis

ATAC-seq was performed as described (Buenrostro et al. 2015) except for the cell preparation steps. To prepare nuclei, 100,000 cells were resuspended in 50 µL of cold lysis buffer (10 mM Tris·Cl at pH 7.4, 10 mM NaCl, 3 mM MgCl_2_, 0.1% [v/v] Igepal CA-630) and incubated for 15 min on ice. The supernatant was discarded after centrifugation, and nuclei were used for transposition reaction immediately. The data were analyzed in the same way as ChIP-seq data. Formaldehyde-assisted isolation of regulatory elements (FAIRE) was performed as described and analyzed with qPCR (Simon et al. 2013).

### RNA-seq analysis

Total RNA was prepared by using the RNeasy minikit (Qiagen, 74104). mRNA was enriched by using oligo dT magnetic beads for library preparation. The paired-end reads were mapped to the mouse reference genome (mm9) using Bowtie2 (version 2.3.2) and TopHat2 (2.0.13) (Kim et al. 2013). Cufflinks (version 2.2.1) (Trapnell et al. 2012) was used to assemble the mapped reads and define differential expression genes (*P*-value < 0.01; fold change > 2). The gene list was further filtered by removing genes with low expression levels (fragments per kilobase per million reads [FPKM] < 1) in both 0-h and pro-B-cell samples. The genes bound by EBF1 within ±25 kb of TSSs were identified as EBF1-regulated genes. Short Time-series Expression Miner (STEM) was used for the clustering of EBF1-regulated genes (Ernst and Bar-Joseph 2006). To identity EBF1-regulated genes, all EBF1-occupied sites that were involved in ±25 kb of TSSs of differential expression genes were considered as functional regulatory elements. The EBF1-occupied sites that are associated with more than one gene were considered as the regulatory element of their nearest genes. The differential expression genes that had at least one EBF1-bound regulatory element within ±25 kb of TSSs were identified as EBF1-regulated genes. For the genes that are bound by EBF1 at multiple sites within ±25 kb of TSSs, all of the EBF1-occupied sites were considered for epigenetic status analysis.

### CRISPR–Cas9 mutagenesis

Guide RNAs (gRNAs) were designed with an online tool (Zhang laboratory, Massachusetts Institute of Technology) and cloned into pSpCas9 (BB)-2A-GFP (PX458; Addgene plasmid 48138; kindly provided by Feng Zhang). For knock-in of mutations, we transfected the template DNA (Ultramer DNA oligonucleotides purchased from Integrated DNA Technologies) with the desired mutation together with the plasmid that could express Cas9 and single gRNA into 38B9 pro-B cells via electroporation. One day after electroporation, GFP-positive cells were sorted and plated into 96-well plates (one cell per well). These single-cell colonies were sequenced, and the homozygous knock-in clones were used for subsequent experiments.

### Data availability

All high-throughput sequencing data presented in this work were uploaded to the Gene Expression Omnibus under one superseries with accession number GSE107242. Individual series can be obtained as follows: GSE107234 (ATAC Cre), GSE107235 (ATAC Tet-on), GSE107236 (EBF1 ChIP Cre), GSE107237 (EBF1 ChIP Tet-on), GSE107238 (Histone ChIP Cre), GSE107239 (Pax5 ChIP Tet-on), GSE107240 (RNA-seq Cre), and GSE107241 (WGBS Cre).

## Supplementary Material

Supplemental Material
