# Supplementary material for: Dynamic EBF1 occupancy directs sequential epigenetic and transcriptional events in B-cell programming
Source: Genes Dev. 2018 Jan 15;32(2):96–111. doi: 10.1101/gad.309583.117 (PMC5830932; doi:10.1101/gad.309583.117)
Supplement: Supplemental Material [file supp_32_2_96__index.html]

Supplemental Material 

# Dynamic EBF1 occupancy directs sequential epigenetic and transcriptional events in B-cell programming

## Supplemental Material

- Supplemental\_Information.docx
- Supplemental\_Table\_S1.xlsx
- Supplemental\_Table\_S2.xlsx
- Supplemental\_Table\_S3.xlsx
