# Supplementary material for: Dynamic EBF1 occupancy directs sequential epigenetic and transcriptional events in B-cell programming
Source: Genes Dev. 2018 Jan 15;32(2):96–111. doi: 10.1101/gad.309583.117 (PMC5830932; doi:10.1101/gad.309583.117)
Supplement: Supplemental Material [file supp_gad.309583.117_Supplemental_Information_.docx]

**Dynamic EBF1 occupancy directs sequential epigenetic and transcriptional**

**events in B cell programming**

Rui Li, Pierre Cauchy, Senthilkumar Ramamoorthy, Sören Boller, Lukas Chavez and

Rudolf Grosschedl

**Supplemental Information**

**Supplemental Inventory:**

Supplemental Figures and Figure Legends:

Supplemental Figure S1 – related to Figure 2

Supplemental Figure S2 – related to Figure 2

Supplemental Figure S3 – related to Figure 3

Supplemental Figure S4 – related to Figure 3

Supplemental Figure S5 – related to Figure 3

Supplemental Figure S6 – related to Figure 4

Supplemental Figure S7 – related to Figures 2A and 5G

Supplemental Materials and Methods

Supplemental References

Legends to Supplemental Tables S1 – S3

Supplemental Tables S1, S2 and S3 (separate excel files)


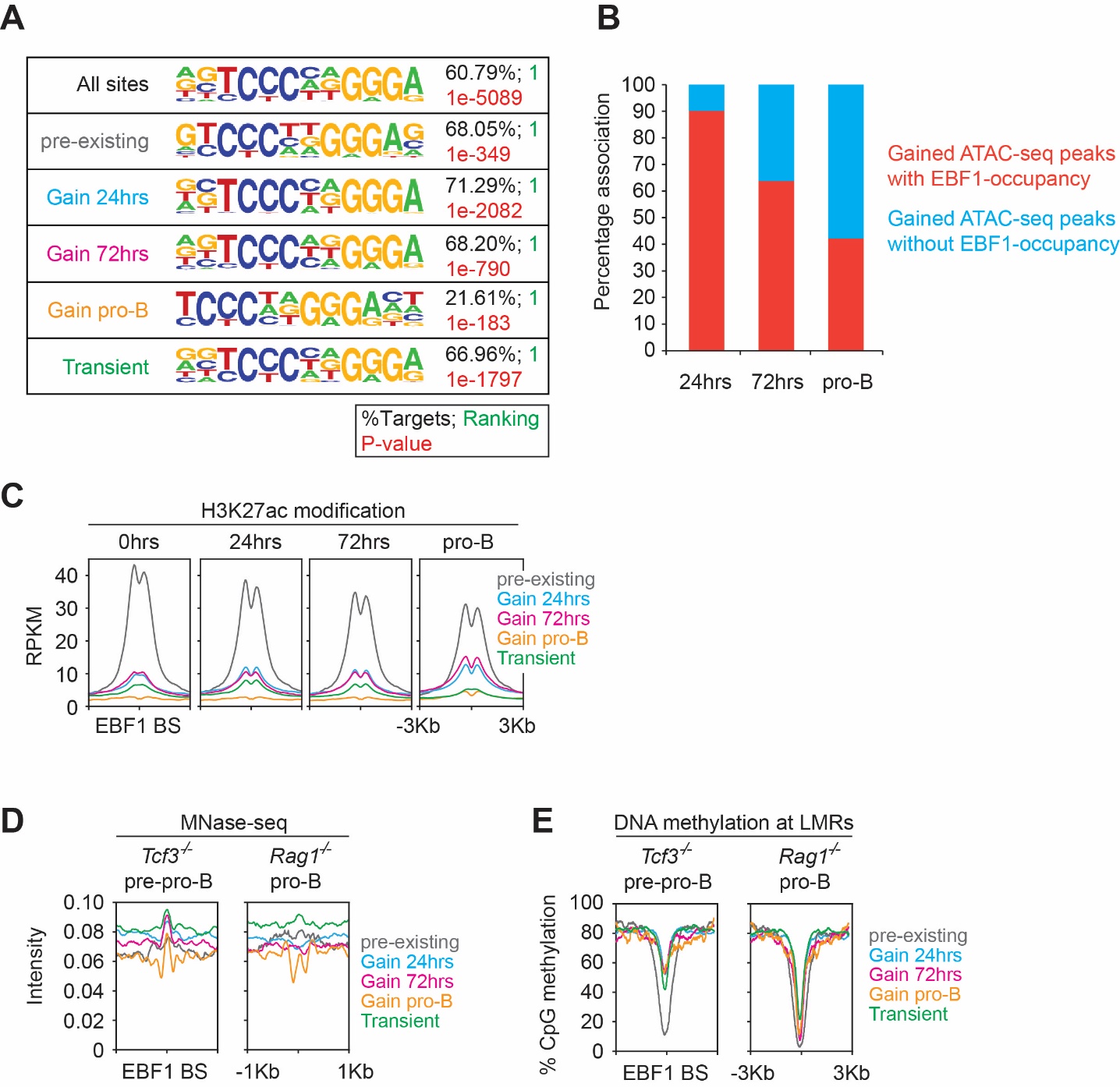


**Figure S1** (related to Figure 2)

(**A**) EBF1-binding motif enriched within ±75 bp distance to the EBF1 peak summit in all peaks and in the five clusters described in Figure 2. The motif ranks, p-value, and percentage of abundance in each individual cluster are shown. (**B**) EBF1 occupancy of the gained ATAC-seq peaks at various time points after EBF1 induction. (**C**) Dynamics of H3K27ac modification centered on EBF1-occupied sites of the clusters described in Figure 2B. (**D**) Average nucleosome profiles within ±1 kb around EBF1-binding sites in pre-pro-B and pro-B cells, using the five clusters defined in Fig. 2. MNase-seq data of *Tcf3^-/-^* pre-pro-B cells and *Rag1^-/-^* pro-B cells were used for this analysis. (**E**) Comparison of EBF1-induced DNA demethylation to published pre-pro-B and pro-B cell whole genome bisulfite sequencing datasets. Average DNA methylation levels (in %) at LMR-overlapping EBF1-binding sites in *Tcf3^-/-^* pre-pro-B cells (left) and *Rag1^-/-^* pro-B cells (right) for all cluster classes, as in Fig. 2D, within ±3kb around EBF1-binding sites. Whole genome bisulfite sequencing data were from (Benner et al. 2015).


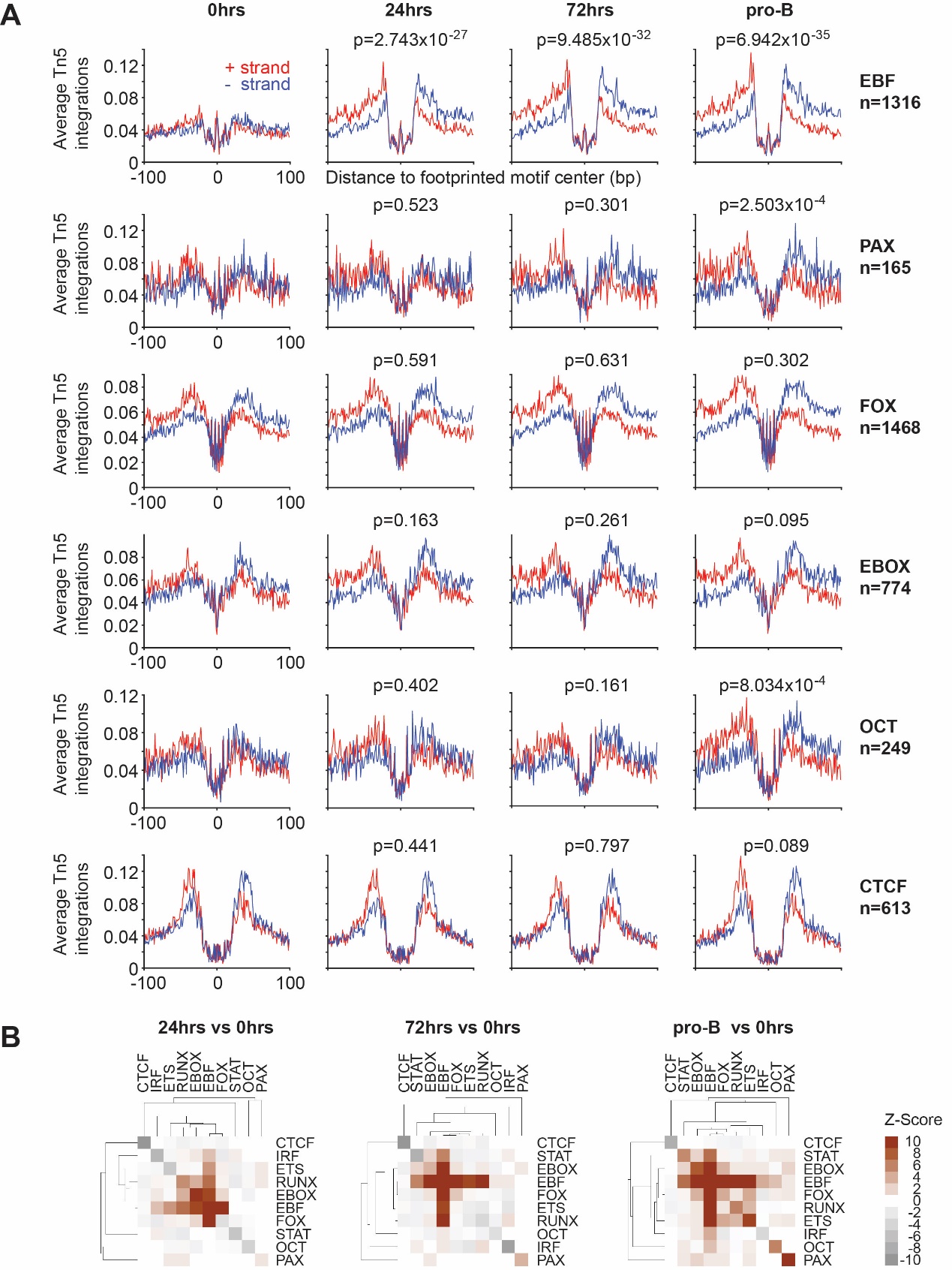


**Figure S2** (related to Figure 2)

Digital genomic footprinting analysis during EBF1-induced B cell programming. (**A**) Average normalized Tn5 insertion profiles around footprinted motifs in merged ATAC peaks at 0 hrs, 24 hrs, 72 hrs after EBF1 induction and at the pro-B cell stage. P-values of F-test indicate significance versus 0 hrs. Sample sizes are indicated on the right. Insertions on the forward and reverse strands are indicated in red and blue, respectively. (**B**) Heatmaps showing footprinted motif co-occurrence enrichment clustering at different time points and stages after EBF1 induction. 24hrs versus 0hrs (left-hand panel); 72hrs versus 0 hrs (middle panel); pro-B stage versus 0 hrs (right-hand panel).


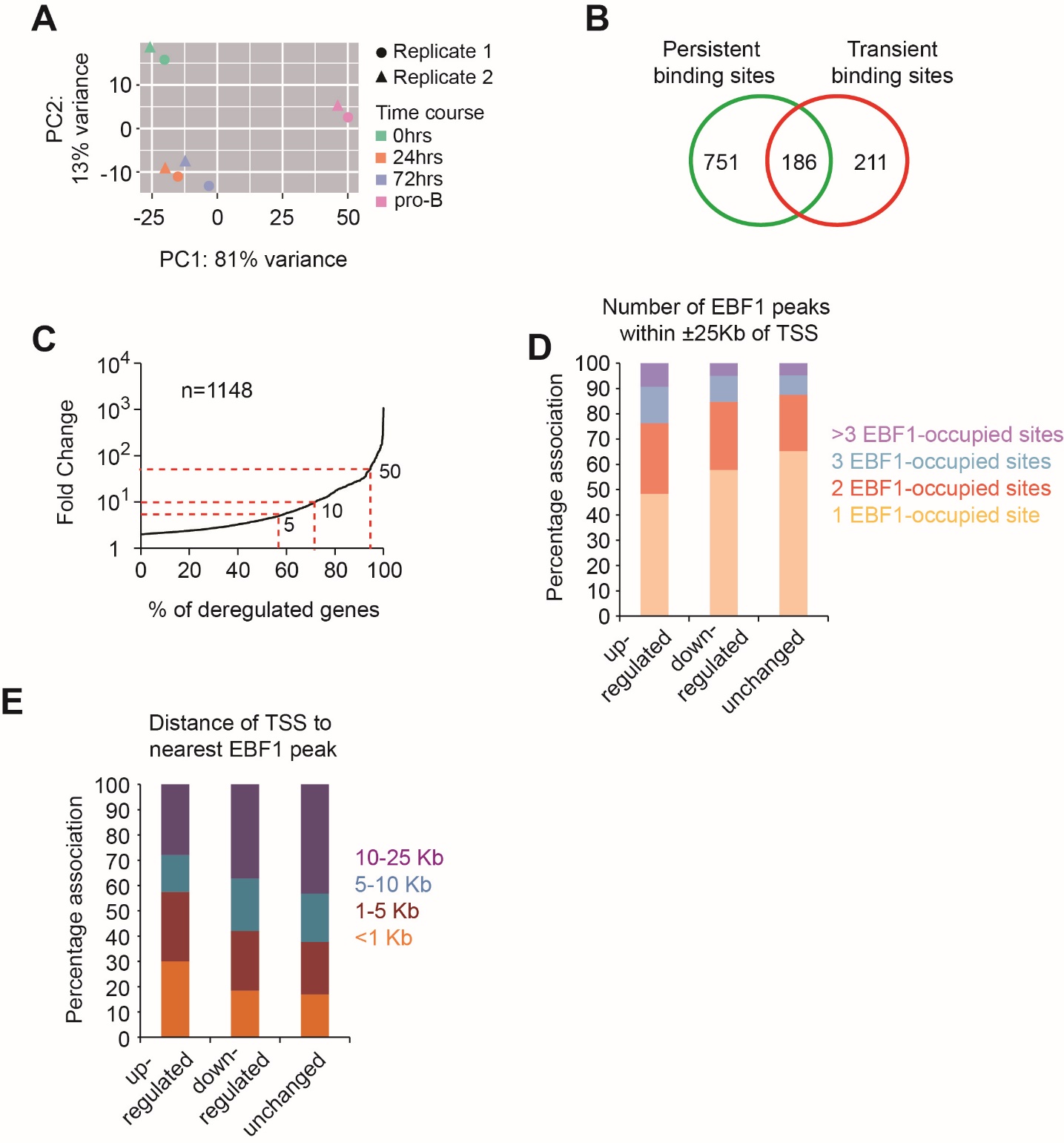


**Figure S3** (related to Figure 3)

(**A**) Principal component analysis of RNA-seq data at different stages of EBF1-induced B cell programming. Two biological replicates are presented with different shape. X-axis and Y-axis represent the top 2 principal components, respectively. (**B**) Venn diagram showing the number of significantly regulated (2-fold change between 0hrs and pro-B, p < 0.01) and expressed (FPKM > 1 in 0hrs or pro-B stage) genes that are persistently and/or transiently occupied by EBF1 within ±25Kb of the transcription start site (TSS). (**C**) The distribution of fold change value between 0hrs and pro-B stages is shown for the 1148 EBF1-regulated genes. The dash red lines represent 5-, 10- and 50-fold change thresholds. (**D**) Number of EBF1 peaks within ±25Kb of TSS is shown for up-regulated, down-regulated and constitutive genes. (**E**) The distance of TSS to the nearest EBF1 peak is shown for up-regulated, down-regulated and constitutive genes. The three groups of genes that are bound by EBF1 within ±25Kb of the transcription start site (TSS) are based on changes of expression between 0 hrs and pro-B cell samples.


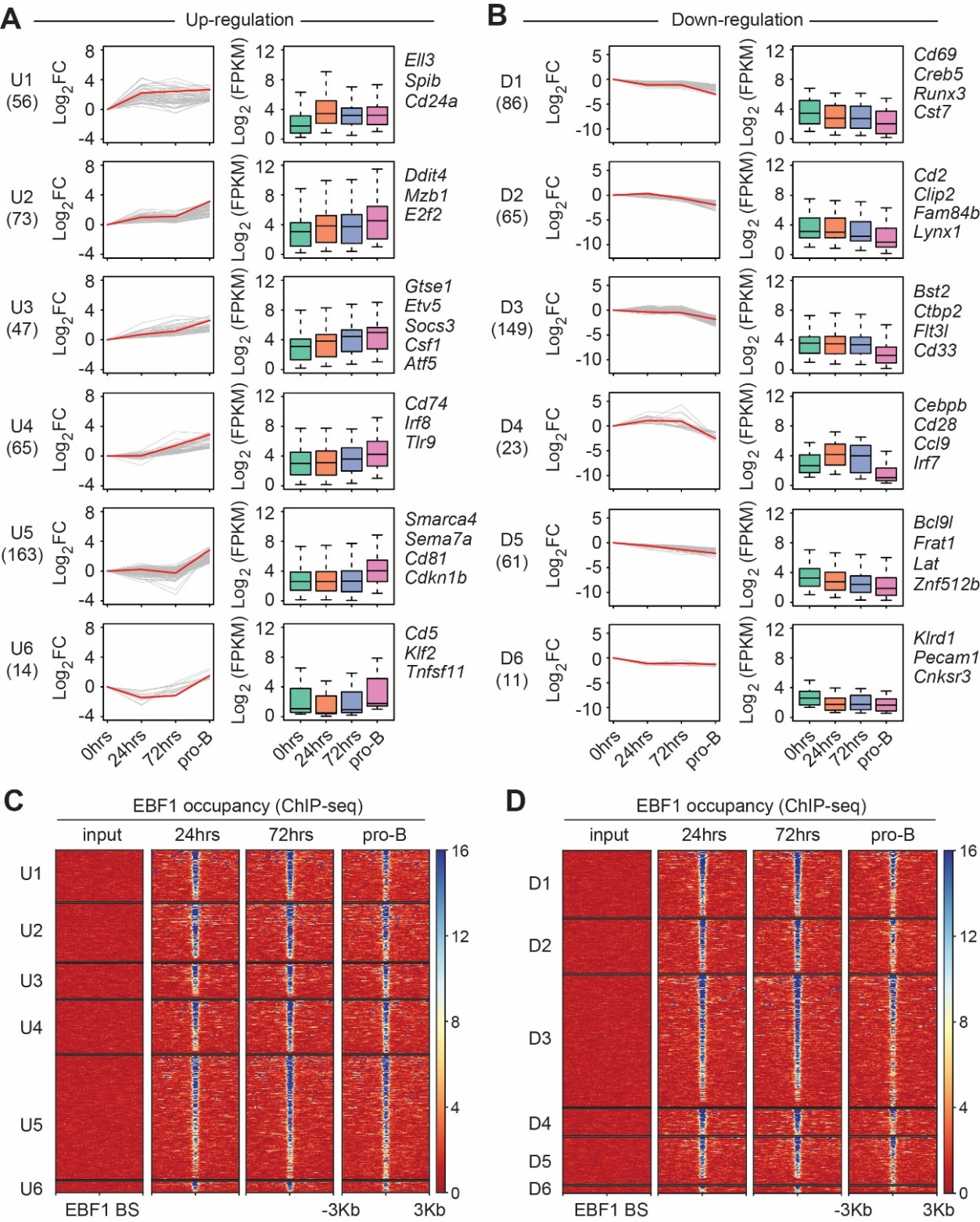


**Figure S4** (related to Figure 3)

(**A** and **B**) Time-resolved analysis of transcript levels of genes containing EBF1-occupied sites within ±25 kb of transcription start sites before and after EBF1 induction. Up-regulated genes (**A**) and down-regulated genes (**B**) that change transcript levels by 2- to 10-fold are shown. Genes are organized into different clusters based on their expression pattern by using Short Time-series Expression Miner (Ernst and Bar-Joseph, 2006). Line plots (left panels) and box plots (right panels) are used to show fold changes (log2 scale) and absolute expression levels, respectively. Representative genes of each cluster are listed on the right. In each line plot, one representative gene is highlighted in red. FC, fold change; FPKM, Fragments Per Kilobase per Million reads. (**C** and **D**) Dynamics of EBF1 occupancy around ±3 kb of EBF1 peaks that are associated with up-regulated genes (**C**) and down-regulated genes (**D**). Clusters correspond to those of the RNA-seq analysis.


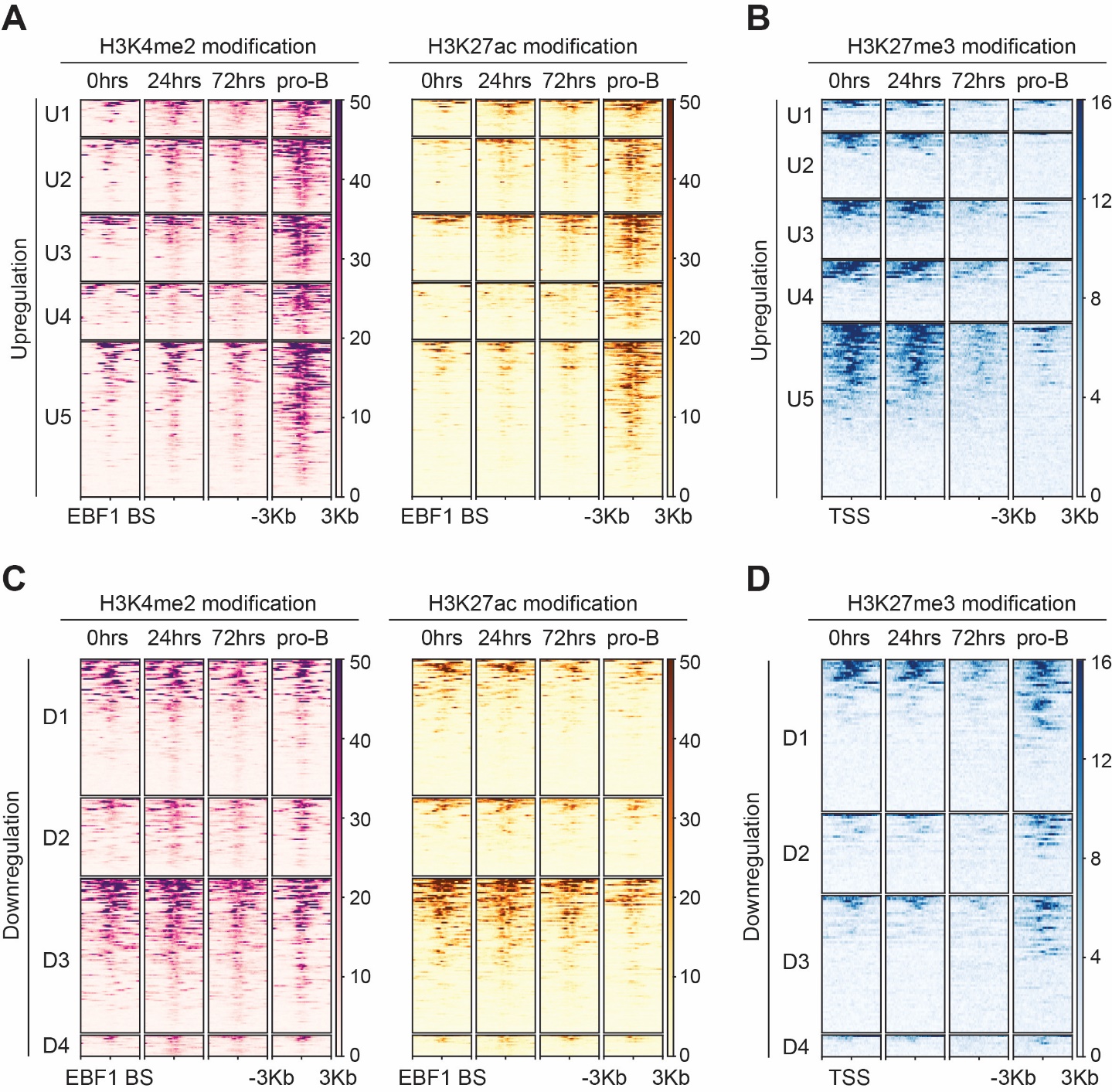


**Figure S5** (related to Figure 3)

(**A**) Dynamics of H3K4me2 and H3K27ac modification around ±3 kb of EBF1-occupied sites associated with genes that are upregulated after EBF1 and grouped into the clusters described in Figure 3. (**B**) Dynamics of H3K27me3 modification around ±3 kb of transcription start sites of genes that are upregulated during EBF1-induced B cell programming and grouped into the clusters described in Figure 3. (**C**) Dynamics of H3K4me2 and H3K27ac modification around ±3 kb of EBF1 binding sites associated with downregulated genes. (**D**) Dynamics of H3K27me3 modification around ±3 kb of transcription start sites of downregulated genes.


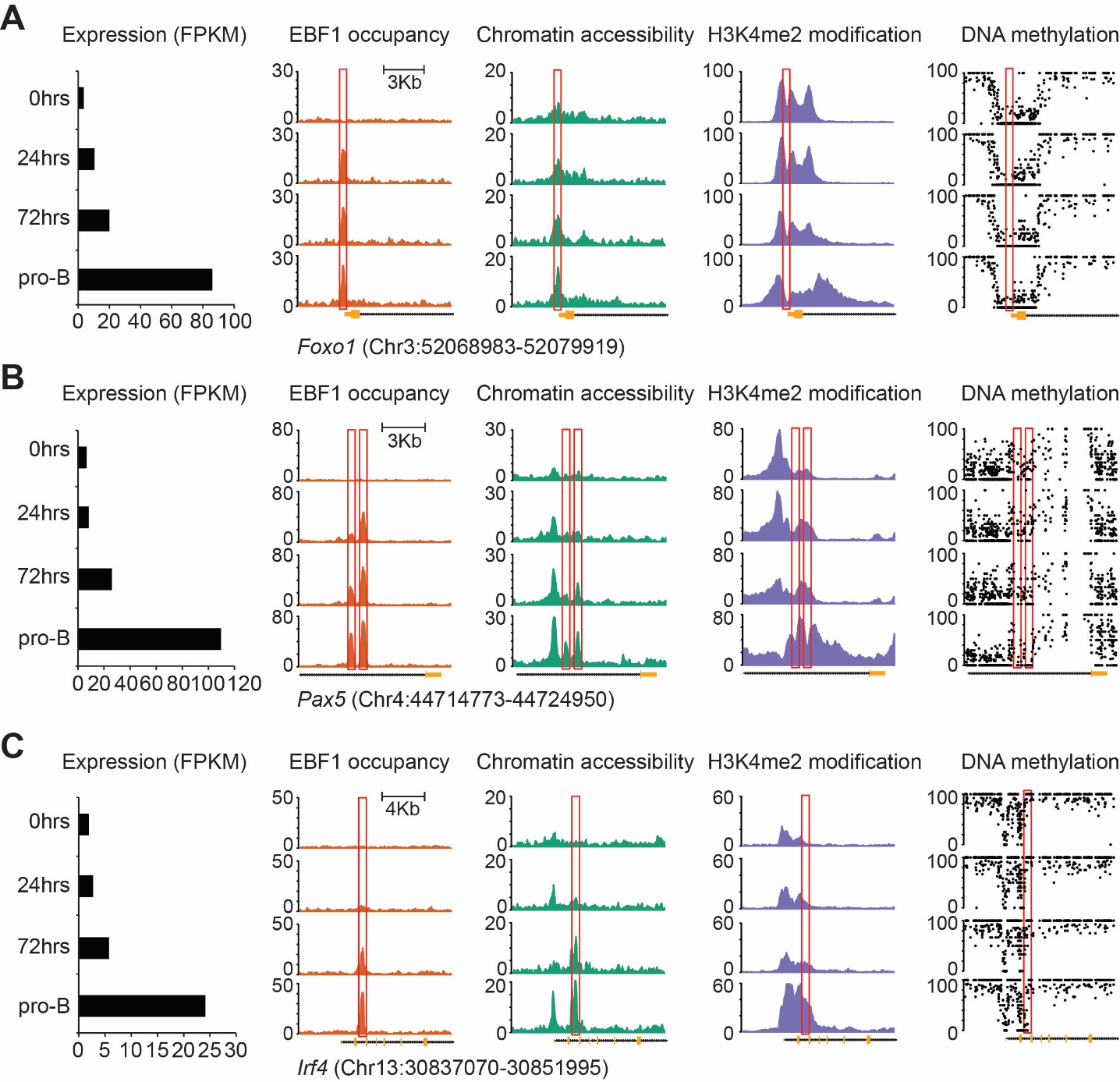


**Figure S6** (related to Figure 4)

Gene-specific analysis of the dynamics of RNA expression, EBF1 occupancy, chromatin accessibility, H3K4me2 modification and DNA methylation after EBF1 induction. Representative genes include (**A**) Foxo1: cluster U3, (**B**) Pax5: cluster U4 and (**C**) Irf4: cluster U4. The positions of EBF1-bound sites are highlighted with red boxes. The scale in the y-axis represents RPKM values in ChIP-seq and ATAC-seq tracks and % in the DNA methylation tracks, in which each black dot represents one CpG.


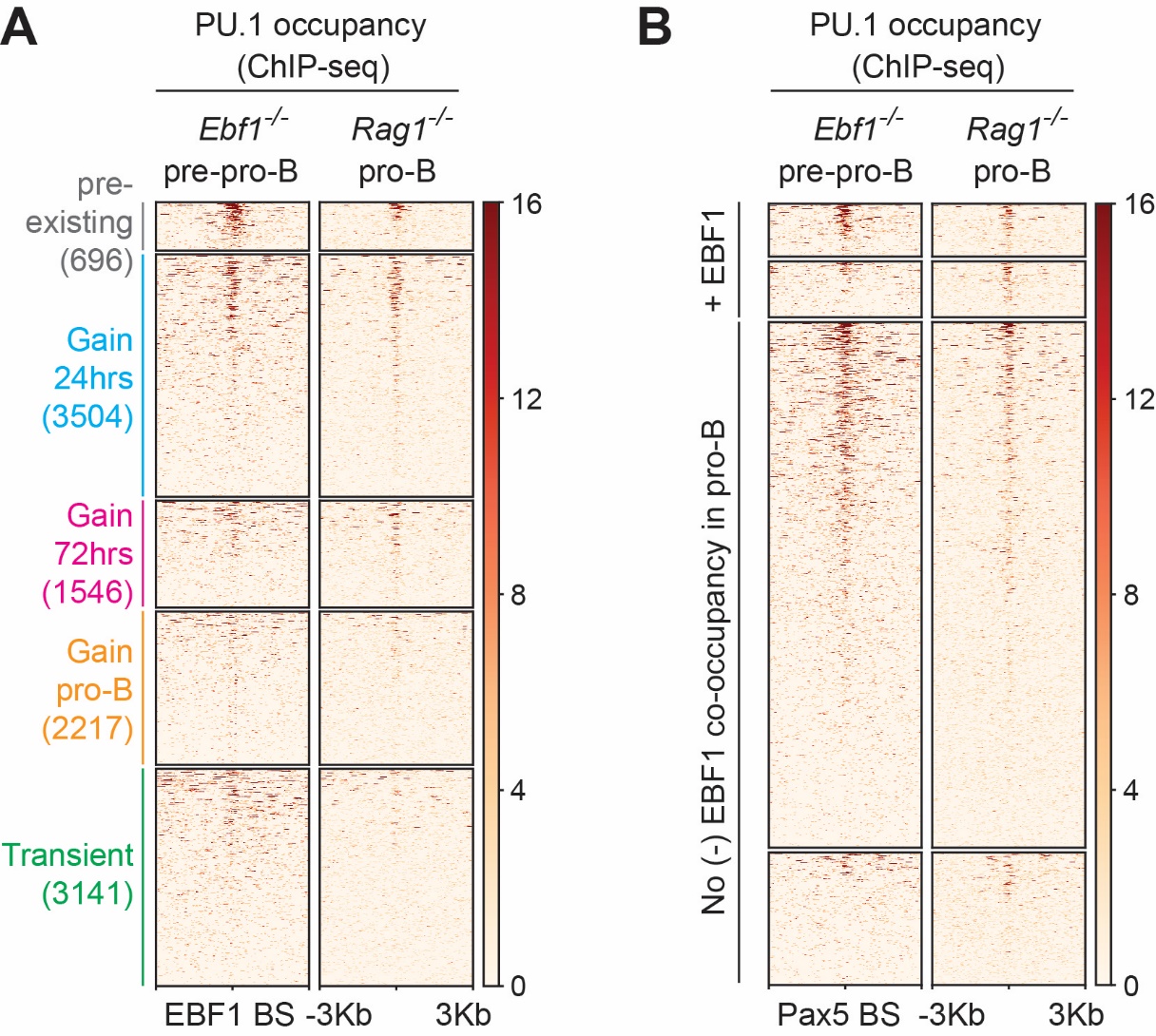


**Figure S7** (related to Figures 2A and 5G)

Analysis of PU.1 occupancy in regions centered around ±3kb of EBF1-bound sites (A) or Pax5-bound sites (**B**) and grouped into clusters described in Fig. 2B and 5H. (**A**) PU.1 occupancy at EBF1-binding sites in the clusters described in Figure 2A in *Ebf1^-/-^* pre-pro-B cells and *Rag1^-/-^* pro-B cells. (**B**) PU.1 occupancy at Pax5-binding sites in the clusters described in Figure 5G in *Ebf1^-/-^* pre-pro-B cells and

*Rag1^-/-^* pro-B cells. The PU.1 ChIP-seq data in *Ebf1^-/-^* pre-pro-B cells and *Rag1^-/-^* pro-B cells were obtained from previously published datasets (Heinz et al. 2010).

**Supplemental Materials and Methods**

*Primers used for qRT-PCR analysis*

| Name | Sequence |
| --- | --- |
| Cd79a For | AACCGCATCATCACAGCAGAAGG |
| Cd79a Rev | GGTTCAGGCCCTCATAGAGATTTTCAT |
| Cd19 For | CTGACCATCGAGAGGCACGT |
| Cd19 Rev | GAGCCACACTGCTGACCTTG |
| Irf4 For | AGATTCCAGGTGACTCTGTGCTTTGGTGA |
| Irf4 Rev | GTAGCCCCTCAGGAAATGTCCAGTGTTTT |
| Ash2l For | CTGGTCCCAGCCCTTAGGTAACC |
| Ash2l Rev | TCCGGCAGTGACTTGGCTGTC |

*Genomic primers (ChIP- and FAIRE-qPCR):*

| Name | Sequence |
| --- | --- |
| Cd79a promoter For | TCGCCAGGATCCTTTCTCAG |
| Cd79a promoter Rev | TATGTGTGGGCTCTGAGTGG |
| Cd19 -2.0Kb For | GTGTGCGCATATCTTTCACTGATGAC |
| Cd19 -2.0Kb Rev | AGTAGAGATAGGCCTGGGACTCAACTG |
| Irf4 +2.1Kb For | GTTTTGTTTCTTTGATTGGGCACCTC |
| Irf4 +2.1Kb Rev | GTCAAAGCTACTCAAAGAAAGAAAATCAG |

*CRISPR-Cas9 oligos:*

| Name | Sequence |
| --- | --- |
| Cd79a gRNA cloning For | CACCGAGAGAGACTCAAGGGAATTG |
| Cd79a gRNA cloning Rev | AAACCAATTCCCTTGAGTCTCTCTC |
| Template for mutation | AGGCCTTGAACCACCCTCTCCCCGACCCCACGCACTAGAGAGAGACTCAACCCAAGCTTTGCCAGCCCAGGTGCAGGGCAGTTCCTCTCCACTCAGAGCCCAC |

*Digital genomic footprinting*

To attain a read depth >100 million reads, replicates of ATAC-Seq experiments were first merged at the bam file level via samtools merge ([Li et al. 2009](#_ENREF_5)) and subsequently sorted and indexed using samtools sort and samtools index, respectively. Peak calling on merged files was performed using MACS2 ([Zhang et al. 2008](#_ENREF_12)). Resulting narrowpeak files were converted to the bed3 format using the bash command cut -f3-. Digital genomic footprinting was performed via the wellington_footprints command of the Wellington package ([Piper et al. 2013](#_ENREF_8)) on bed3-converted peaks from combined replicates, using sorted and indexed bam files, with the ATAC -A switch which results in the reference being shifted by -4bp and +5bp for the forward and reverse strands, respectively, due to the double, 9bp-spaced nature of insertions by Tn5 dimers ([Buenrostro et al. 2013](#_ENREF_1)). Footprints having a p-value lower than or equal to 10^-10^ were retained. For average insertion profiles per motif, the union of all footprints was first computed by merging all footprints using the following command: cat <footprints> | bedtools sort -i - | bedtools merge –i –d 20. Motifs were mapped to footprints via the Homer annotatePeaks ([Heinz et al. 2010](#_ENREF_3)) command using -m -mbed <bed file> -size given switches. Average insertion profiles were obtained using the dnase_average_profiles command of the Wellington package, using -n -A as parameters. Co-occurrence enrichment clustering analyses were performed exactly as previously described for footprints ([Obier et al. 2016](#_ENREF_7)), using footprints and motifs from this study.

*Bisulfite-seq and MNase-seq analysis*

Genomic DNA was prepared using DNeasy Blood & Tissue Kit (Qiagen, 69504). Bisulfite conversion, library preparation and deep sequencing were performed by Genomics and Proteomics Core Facility in German Cancer Research Center (DKFZ). To optimize read qualities, reads were adapter-, quality- and end- trimmed using TrimGalore version 0.3.7 (https://www.bioinformatics.babraham.ac.uk/projects/trim_galore/), a wrapper around cutadapt ([Martin 2011](#_ENREF_6)), using --paired –phred 33 -q 20 -e 0.15 --clip-R1 14 --clip-R2 15 --retain-unpaired --fastqc --three-prime-clip-R1 20 --three-prime-clip-R2 -a <index-specific adapter> -a2 <index-specific adapter> as parameters. Alignment to the mm9 genome was performed via bismark version 0.16.3 ([Krueger and Andrews 2011](#_ENREF_4)) using the following steps: 1) forward and reverse bisulfite-converted mm9 genomes were prepared via bismark_genmome_preparation using --path_to_bowtie </path/to/bowtie2/> as parameters 2) sequential alignment to the bisulfite-converted and original mm9 genomes was obtained using the main bismark program, with --genome </path/to/original/and/bisulfite/converted/mm9/genomes/> -1 <read1> -2 <read2>. Methylation calls and coverages were retrieved using bismark_methylation_extractor using --gzip --bedGraph <bam_file> as parameters. Un- and low-methylation region calls (UMRs and LMRs, respectively) were computed via the MethylSeekR package version 1.16.0 ([Burger et al. 2013](#_ENREF_2)) for R. Bismark coverages were first converted to MethylSeekR compatible tables by, using bismark coverage tables, retrieving columns 1 and 2 (chromosome, position), adding columns 5 and 6 together (respectively the number of read methylated and unmethylated cytosines at each position), in order to obtain the total number of cytosines read at a given position, becoming column 3 of the newly-created MethylSeekR compatible object, and retrieving column 5, becoming column 4 (number of methylated cytosines of the new object. Detection of UMRs and LMRs was carried out using default parameters, first identifying partially methylated domains (PMDs), excluding CpG islands, and training the LMR and UMR model on chromosome 19. UMRs and LMRs were exported as bed files used for further analysis. LMRs were intersected with EBF1 binding sites of the above-defined clusters as well as total clusters (total consistent, total transient sites) using bedtools intersect ([Quinlan and Hall 2010](#_ENREF_9)), further depleting of CpG islands via bedtools intersect -v in the case of partial overlap with EBF1 sites but not LMR sites. Scatterplots were obtained using the smoothScatter function of R, using a bandwith of 3, on average methylation counts ±150bp of the EBF1 summit. For average methylation profiles, bedGraph files were converted to the bigwig format using the UCSC Genome Browser bedGraphToBigWig utility, and used to generate methylation tables corresponding to clusters intersecting LMRs via the computeMatrix function of DeepTools ([Ramirez et al. 2016](#_ENREF_10)), using reference-point --referencePoint center -b 3000 -a 3000 -R <bed files> -S <bigwig files> as parameters. Average methylation values were smoothed using a moving average of bin size 200 bp. For previously published WGBS data in pre-pro and pro-B cells, mm9 bigwig files were obtained directly from GEO as accessions GSM1867946 and GSM1867947, and processed as they were using deeptools computeMatrix and deeptools plotProfile to compute and display average profiles.

For MNase-Seq, fastq and mm9 eland files were obtained (Claudia Bossen, personal communication) and subsequently processed using macs2 callpeak 2.1.0 using --nomodel --extsize 146 --g mm --keep-dup all --B --trackline --SPMR, to in order to obtain normalized bedGraph coverage tracks reflecting mononucleosomal fragment sizes. BedGraph coverage were subsequently converted to the bigwig format via the bedGraphToBigWig utility and plugged in to deeptools computeMatrix and deeptools plotProfile to compute and display average profiles.

**SUPPLEMENTAL REFERENCES**

Benner C, Isoda T, Murre C. 2015. New roles for DNA cytosine modification, eRNA, anchors, and superanchors in developing B cell progenitors. *Proc Natl Acad Sci U S A* **112**: 12776-12781.

Bossen C, Murre CS, Chang AN, Mansson R, Rodewald HR, Murre C. 2015. The chromatin remodeler Brg1 activates enhancer repertoires to establish B cell identity and modulate cell growth. *Nature immunology* **16**: 775-784.

Buenrostro JD, Giresi PG, Zaba LC, Chang HY, Greenleaf WJ. 2013. Transposition of native chromatin for fast and sensitive epigenomic profiling of open chromatin, DNA-binding proteins and nucleosome position. *Nature methods* **10**: 1213-1218.

Burger L, Gaidatzis D, Schubeler D, Stadler MB. 2013. Identification of active regulatory regions from DNA methylation data. *Nucleic acids research* **41**: e155.

Heinz S, Benner C, Spann N, Bertolino E, Lin YC, Laslo P, Cheng JX, Murre C, Singh H, Glass CK. 2010. Simple combinations of lineage-determining transcription factors prime cis-regulatory elements required for macrophage and B cell identities. *Molecular cell* **38**: 576-589.

Krueger F, Andrews SR. 2011. Bismark: a flexible aligner and methylation caller for Bisulfite-Seq applications. *Bioinformatics* **27**: 1571-1572.

Li H, Handsaker B, Wysoker A, Fennell T, Ruan J, Homer N, Marth G, Abecasis G, Durbin R, Genome Project Data Processing S. 2009. The Sequence Alignment/Map format and SAMtools. *Bioinformatics* **25**: 2078-2079.

Martin M. 2011. Cutadapt removes adapter sequences from high-throughput sequencing reads. *2011* **17**.

Obier N, Cauchy P, Assi SA, Gilmour J, Lie ALM, Lichtinger M, Hoogenkamp M, Noailles L, Cockerill PN, Lacaud G et al. 2016. Cooperative binding of AP-1 and TEAD4 modulates the balance between vascular smooth muscle and hemogenic cell fate. *Development* **143**: 4324-4340.

Piper J, Elze MC, Cauchy P, Cockerill PN, Bonifer C, Ott S. 2013. Wellington: a novel method for the accurate identification of digital genomic footprints from DNase-seq data. *Nucleic acids research* **41**: e201.

Quinlan AR, Hall IM. 2010. BEDTools: a flexible suite of utilities for comparing genomic features. *Bioinformatics* **26**: 841-842.

Ramirez F, Ryan DP, Gruning B, Bhardwaj V, Kilpert F, Richter AS, Heyne S, Dundar F, Manke T. 2016. deepTools2: a next generation web server for deep-sequencing data analysis. *Nucleic acids research* **44**: W160-165.

Revilla IDR, Bilic I, Vilagos B, Tagoh H, Ebert A, Tamir IM, Smeenk L, Trupke J, Sommer A, Jaritz M et al. 2012. The B-cell identity factor Pax5 regulates distinct transcriptional programmes in ear ly and late B lymphopoiesis. *The EMBO journal* **31**: 3130-3146.

Zhang Y, Liu T, Meyer CA, Eeckhoute J, Johnson DS, Bernstein BE, Nusbaum C, Myers RM, Brown M, Li W et al. 2008. Model-based analysis of ChIP-Seq (MACS). *Genome biology* **9**: R137.

**Legends to Supplemental Tables**

**Table S1.** RNA-seq data of more than 10-fold deregulated genes. Genes belong to different clusters are grouped into separated worksheets and ranked by fold change. The normalized expression of each gene is shown as FPKM value. EBF1 occupancy, Pax5 co-occupancy and the Pax5 regulation patterns are presented for each gene. Pax5 regulation pattern is defined by comparing the expression level between *Pax5*-deficient and wild type pro-B cells ([Revilla et al. 2012](#_ENREF_11)). The data of this table correspond to Figure 3A and 3B.

**Table S2.** RNA-seq data of 2-10-fold deregulated genes. Genes belong to different clusters are grouped into separated worksheets and ranked by fold change. The normalized expression of each gene is shown as FPKM value. EBF1 occupancy, Pax5 co-occupancy and Pax5 regulation patterns are presented for each gene. Pax5 regulation pattern is defined by comparing the expression level between *Pax5*-deficient and wild type pro-B cells ([Revilla et al. 2012](#_ENREF_11)). The data of this table correspond to Figure S4A and S4B.

**Table S3.** EBF1-dependent Pax5-occupied sites. The location and nearest gene of each Pax5 peak are presented. The data of this table correspond to Figure 5G.
